# A preclinical study of allogeneic CD19 chimeric antigen receptor double‐negative T cells as an off‐the‐shelf immunotherapy drug against B‐cell malignancies

**DOI:** 10.1002/cti2.70022

**Published:** 2024-12-24

**Authors:** Dan Wang, Liuyang Wang, Shuai Liu, Jianjun Tong, Honglin Zhu, Man Xu, Xiancai Li, Zhiqiang Xiang, Qinghua Sun, Hengcai Wang, Yuli Wang, Shuyang Wang, Liming Yang

**Affiliations:** ^1^ Wyze Biotech Co. Ltd Zhongshan Guangdong China; ^2^ The Center for Drug Safety Evaluation and Research, Shanghai Institute of Materia Medica Chinese Academy of Sciences (CDSER/SIMM) Shanghai China

**Keywords:** allogeneic CD19‐CAR‐DNTs, B‐cell malignancies, cytotoxicity and safety, off‐the‐shelf immunotherapy

## Abstract

**Objectives:**

To evaluate the manufacturability, efficacy and safety of allogeneic CD19 chimeric antigen receptor double‐negative T cells (CD19‐CAR‐DNTs) as an off‐the‐shelf therapeutic cell product.

**Methods:**

A membrane proteome array was used to assess the off‐target binding of CD19‐CAR. DNTs derived from healthy donors were transduced with lentiviral vectors encoding the CD19‐CAR. The manufacture of the CD19‐CAR‐DNTs was under GMP conditions, and their surface molecule expression patterns were characterised using flow cytometry. We investigated the off‐the‐shelf potential of CD19‐CAR‐DNTs by evaluating the cryopreserved CD19‐CAR‐DNTs in terms of cell viability as well as their cytotoxicity against various CD19^+^ target cell lines and primary patient blasts *in vitro.* We evaluated the persistence and safety of the cryopreserved CD19‐CAR‐DNTs in xenograft models *in vivo*.

**Results:**

GMP‐grade CD19‐CAR‐DNTs were manufactured and cryopreserved for use in advance. The cryopreserved CD19‐CAR‐DNTs maintain their viability and antitumor activity against various CD19^+^ target cell lines and primary patient blasts. These cells significantly prolonged the survival in Raji‐Luc‐xenografted NOG mice. Multiple infusions of the cells can further augment their efficacy. Remarkably, following a single infusion in mice, CD19‐CAR‐DNTs rapidly got distributed among well‐perfused organs initially, and progressively spread to most tissues, peaking at Day 43. In toxicity studies, CD19‐CAR‐DNTs significantly reduced tumor burden and ameliorated tissue damage in tumor‐bearing NOG mice. Critically, no immunotoxicity or graft versus host disease was observed in non‐tumor‐bearing NOG mice.

**Conclusions:**

The allogeneic CD19‐CAR‐DNTs fulfil the requirements of an off‐the‐shelf therapeutic cell product, offering a promising new approach to the treatment of haematological malignancies.

## Graphical abstract

In this study, we found that a novel off‐the‐shelf CD19 double‐negative T cell (CD19‐CAR‐DNT) therapy demonstrated significant antitumor activity and safety *in vitro* and *in vivo*. GMP‐grade CD19‐CAR‐DNTs maintained efficacy after cryopreservation and significantly extended survival in tumor models. No toxicity or graft versus host disease was observed, offering a new strategy for the treatment of haematologic malignancies.

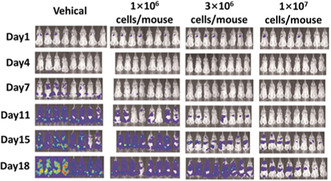

## Introduction

Chimeric antigen receptor T cells (CAR‐Ts) have shown remarkable efficacy in cancer therapy.^1^, ^2^, ^3^, ^4^, ^5^, ^6^, ^7^ However, the CAR T‐cell products that have been approved for marketing so far are prepared with patient‐derived T cells, with limitations that not only hinder life‐saving treatment access by patients a but also challenge their use as a standard‐of‐care treatment.^8^, ^9^, ^10^ Additionally, there have been reports of viral transduction of patients’ residual tumor cells with CAR‐encoding genes.^11^ The utilisation of immune cells obtained from healthy donors as a source for CAR T‐cell production offers a potential solution to the aforementioned issues.^9^, ^12^ The standardised manufacturing of CAR T‐cell products from healthy donors is anticipated to effectively address issues related to accessibility and high cost. Nevertheless, there remain obstacles in the development of allogeneic CAR T‐cell therapy because of a risk of graft versus host disease (GvHD) despite the knockout of T‐cell receptor (TCR) or human leukocyte antigens (HLA) genes.^3^

Double‐negative T cells (DNTs) that constitute approximately 1–10% of human peripheral blood mononuclear cells (PBMCs) are mature T cells that express CD3, αβ‐TCR or γδ‐TCR but lack CD4 and CD8 molecules.^13^, ^14^, ^15^ Notably, we have observed that *ex vivo* expanded DNTs from healthy donors predominantly express γδ‐TCR, including both Vδ1 and Vδ2 TCR cells, with Vδ2 cells being the predominant subtype. These cells exhibit both innate and adaptive immune features, recognising tumor cells through natural cell receptors (NCRs) such as NKG2D and DNAM‐1, independent of major histocompatibility complex molecules. DNTs secrete a range of cytotoxic molecules and cytokines to eliminate tumor cells, demonstrating potent antitumor effects against various malignancies, including acute myeloid leukaemia (AML), lymphoma, non‐small‐cell lung cancer and pancreatic cancer.^14^, ^16^, ^17^, ^18^, ^19^, ^20^, ^21^ Importantly, allogeneic DNTs do not target haematopoietic stem and progenitor cells or interfere their differentiation nor are they cytotoxic to normal allogeneic PBMCs. Infusion of human DNTs has been shown to be safe, not causing GvHD and resistant to host versus graft rejection (HvGR) in preclinical models.^14^, ^22^, ^23^, ^24^, ^25^ Clinical trials have further demonstrated the safety and promising efficacy of allogeneic DNTs in treating relapsed and refractory AML.^26^, ^27^ Therefore, DNTs hold promise as an off‐the‐shelf cellular therapy for the treatment of various tumors.

In this study, we transduced healthy donor‐derived DNTs with a CD19 lentiviral vector and evaluated their feasibility as an off‐the‐shelf immunotherapy drug. *Ex vivo* expanded CD19‐CAR‐DNTs from healthy donors were cryopreserved in a liquid nitrogen tank for at least 9 months without compromising their tumoricidal activity. In a xenograft model of human B‐cell non‐Hodgkin lymphoma (B‐NHL), NOG mice treated with cryopreserved CD19‐CAR‐DNTs exhibited robust tumor growth inhibition (TGI). Furthermore, multiple infusions further enhanced the antitumor activity. CD19‐CAR‐DNTs distributed to various organs, particularly those well‐perfused with blood, proliferated dramatically without causing notable immunotoxicity and GvHD. Collectively, these data support the utilisation of CD19‐CAR‐DNTs as an allogeneic cell therapy (off‐the‐shelf) for the treatment of B‐cell malignancies.

## Results

### Humanised CD19 scFv can specifically target CD19 protein without any off‐target binding

**Figure 1:**
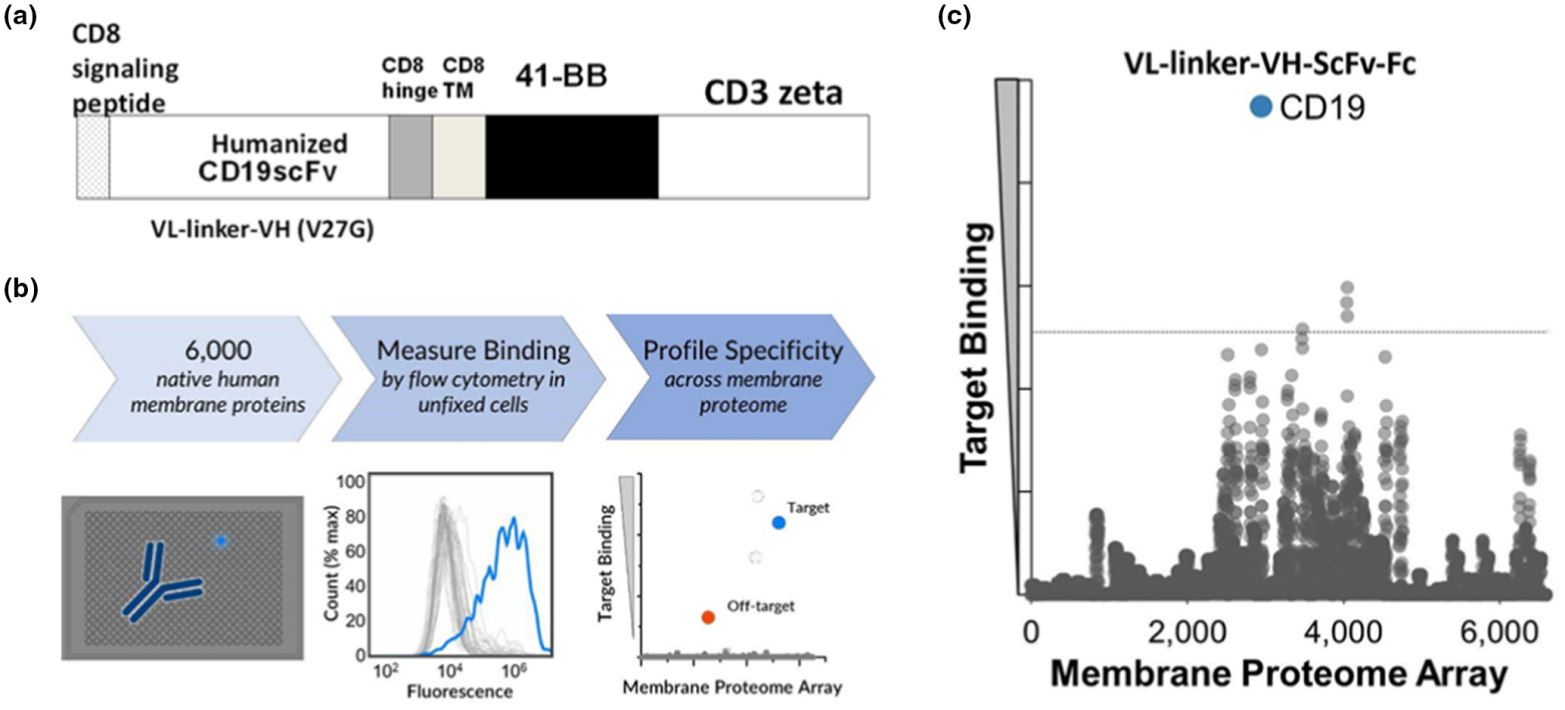
Humanised CD19 scFv exhibits a remarkable specificity in targeting the CD19 protein. **(a)** Schematic diagram of the second‐generation CD19 chimeric antigen receptor (CD19‐CAR), which consists of the scFv combined with CD8α hinge and transmembrane regions fused to the intracellular signalling domains of 4‐1BB and CD3ζ. **(b)** Membrane proteome array overview. **(c)** Each ligand was tested for binding by flow cytometry against the MPA at 20 μg mL^−1^. Binding interactions confirmed in downstream validation studies are displayed in blue, and any proteins that did not pass validation were removed.

We designed CAR consisting of anti‐CD19 scFv and CD8 trans‐membrane domain in tandem with 41BB intracellular signalling domain and CD3ζ (Figure 1a). To investigate the potential for off‐target binding of this humanised CD19‐CAR, we first constructed a recombinant humanised CD19 ScFv antibody (Supplementary figure 1). Subsequently, we employed a membrane proteome array (MPA), a powerful platform for profiling the specificity of antibodies and other ligands that target human membrane proteins (Figure 1b). MPA enables us to determine the target specificity of antibodies and decipher orphan antibody/ligand targets by testing them against a comprehensive collection of human membrane proteins.

The recombinant humanised CD19 scFv antibody was tested for reactivity against over 6000 human membrane proteins, including 94% of all single‐pass, multi‐pass and GPI‐anchored proteins, including GPCRs, ion channels and transporters. As shown in Figure 1c and Table 1, the humanised CD19 scFv antibody specifically targets human CD19 protein with no detectable off‐target binding, demonstrating its high specificity and potential for safe and effective application in adoptive T‐cell therapy.

**Table 1:** Validated final membrane proteome array screen results

Test ligands with no newly identified targets: VL‐linker‐VH‐ScFv‐Fc				
Test ligand name	Screening cell line	Screening concentration, μg mL^−1^	Known target(s)	
			HGNC	Uniprot
VL‐linker‐VH‐ScFv‐Fc	HEK‐293 T	20	CD19	P15391

### DNTs can be stably transduced with a lentiviral vector to express CD19‐CAR and without altering the phenotype of DNTs

DNTs derived from healthy donors show robust cytotoxicity against a broad spectrum of haematologic and solid tumor cell lines, fulfilling the criteria for an off‐the‐shelf universal adoptive T‐cell therapy.^17^, ^18^, ^19^, ^20^, ^21^, ^25^, ^26^, ^27^ As previously shown by Daniel *et al*.,^28^ DNTs are amendable to CAR transduction using a retroviral vector. However, in comparison with retroviral vectors, the utilisation of a self‐inactivating lentiviral vector to attenuate CAR expression has minimised antigen‐independent tonic signalling, while enhancing T‐cell expansion and antitumor function.^29^ Here, we aimed to ascertain whether DNTs could be stably transduced with a lentiviral vector while maintaining the biological characteristics of DNTs.

**Figure 2:**
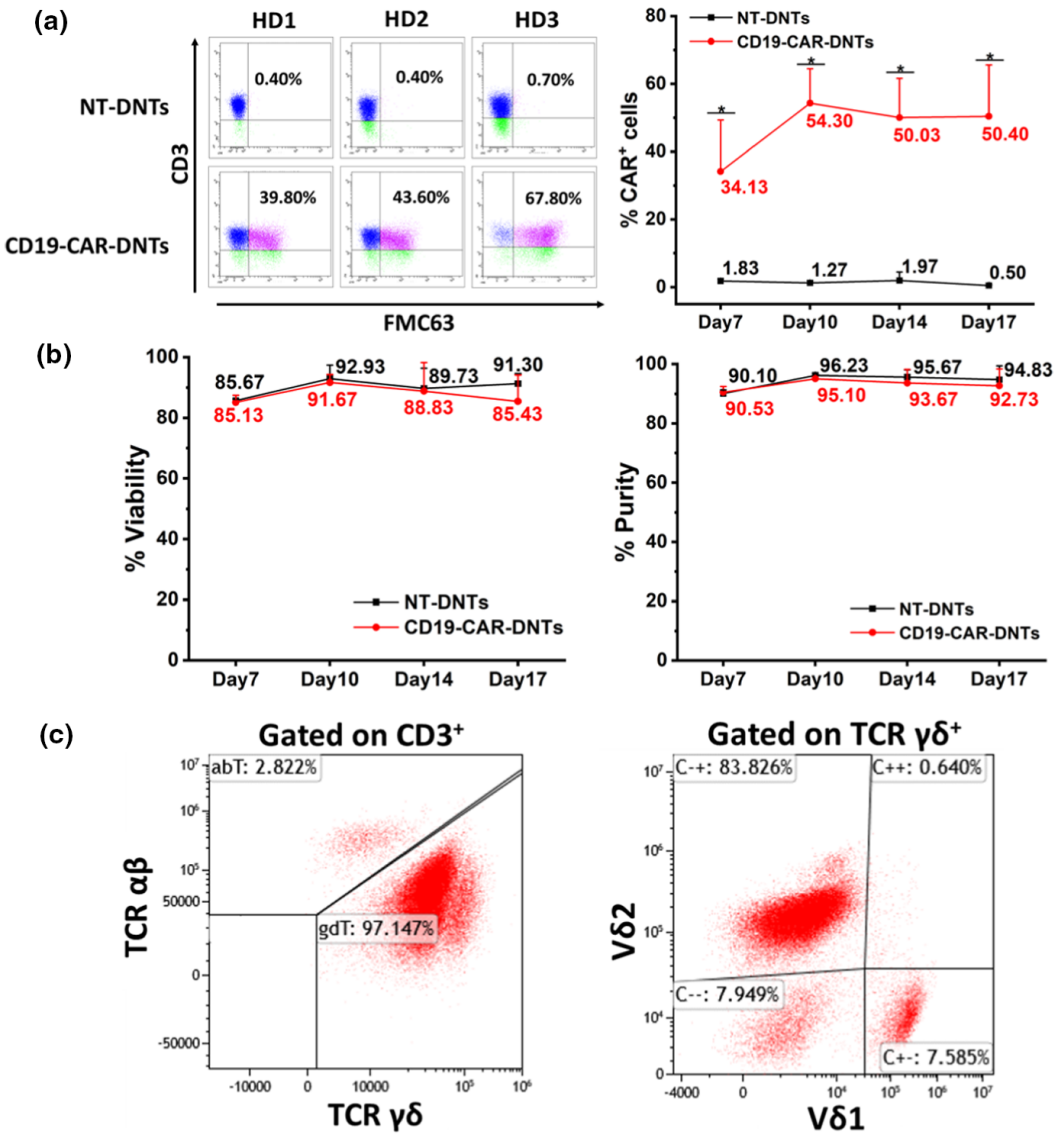
Double‐negative T cells (DNTs) transduced with CD19 chimeric antigen receptor (CD19‐CAR) lentiviral vector retained their innate characteristics and exhibited stable CAR expression over 17 days. **(a)** Left: representative histograms showing anti‐CD3 and anti‐FMC63 (CD19‐CAR) staining on transduced [CD19 chimeric antigen receptor double‐negative T cells (CD19‐CAR‐DNTs)] and non‐transduced DNTs (NT‐DNTs). The numbers indicate the percentage of CD19‐CAR expression on DNTs. Right: CD19‐CAR expression was measured over 17 days. Data are shown as mean ± standard deviation of triplicates and are representative of three independent experiments using three different donors, *P < 0.05 (*t*‐test). **(b)** Viability and purity (CD3^+^CD4^−^CD8^−^) of CD19‐CAR‐DNTs and NT‐DNTs remained high over the 17 days. Data are shown as mean ± SD of triplicates and are representative of three independent experiments using three different donors. **(c)** Expression of αβ‐TCR, γδ‐TCR, Vδ1‐TCR and Vδ2‐TCR on CD19‐CAR‐DNTs was analysed by flow cytometry. The results are representative of three independent experiments. Numbers indicate the percentage of cells in each gate.

As illustrated in Figure 2a, DNTs were successfully transduced by a lentiviral vector with CD19‐CAR, and the CAR expression on DNTs remained stable from Day 10 to Day 17. Notably, the viability and purity (CD3^+^CD4^−^CD8^−^) of CD19‐CAR‐DNTs remained above 85% from Day 7 to Day 17, similar to that of non‐transduced DNTs (NT‐DNTs) (Figure 2b). These findings indicate that DNTs transduced with CD19 lentivirus retain their biological characteristics and viability, comparable to NT‐DNTs. Moreover, the development of a large‐scale manufacturing process for producing clinically relevant numbers of CD19‐CAR‐DNTs is crucial for their potential clinical application. In this regard, we have successfully developed the manufacturing process to expand CD19‐CAR‐DNTs under GMP conditions and achieved a median expansion of 2977.5‐fold (range 1281.2–5068.2) on Day 14 (Supplementary figure 2a). The median transduction efficiency of DNTs was 51.1% (range 43.6–65.5%), and the median viability of CD19‐CAR‐DNTs was 93.0% (range 89.6–94.4%) on either Day 13 or Day 14 of *ex vivo* culture (Supplementary figure 2b,c). Furthermore, the phenotype of CD19‐CAR‐DNT products was analysed by flow cytometry (Figure 2c). It reveals that the transduced cells were primarily composed of αβ‐T and γδ T cells, predominantly γδ T cells (>90%). Specifically, the γδ‐T cell subset was further analysed, and it was found that it comprises Vδ1 and Vδ2 cells, with Vδ2 cells being the most abundant. The CD19‐CAR‐DNTs consist of an average of 93.42% DNTs with CD4^+^/CD8^+^ T cells and NK cells as residue. No B cells or monocytes were detected in the final cell product (Supplementary figure 2d).

### Cryopreserved CD19‐CAR‐DNTs can be stored in liquid nitrogen without compromising their biological functions

**Figure 3:**
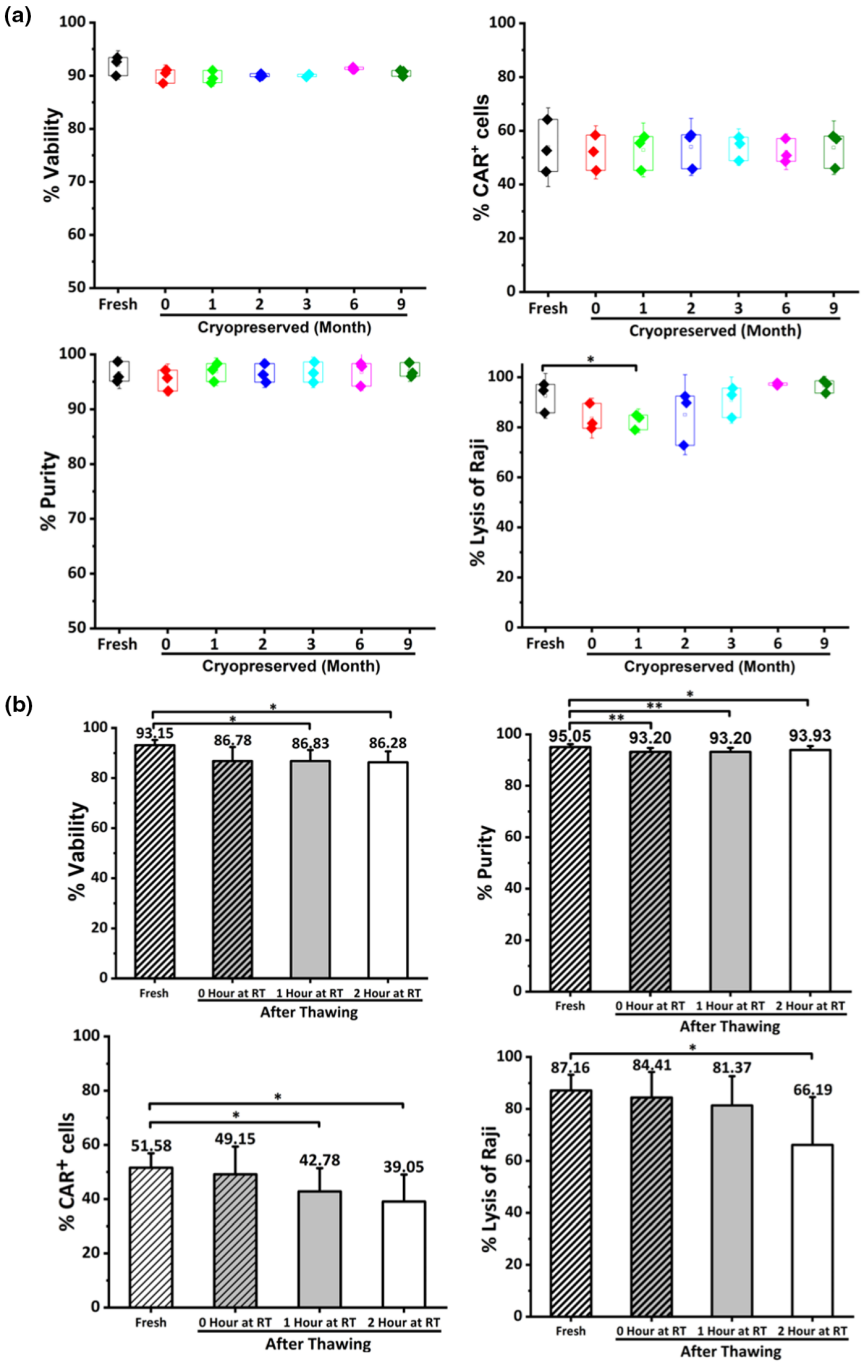
Cryopreservation of CD19 chimeric antigen receptor double‐negative T cells (CD19‐CAR‐DNTs) efficiently preserves their biological functionality. **(a)** CD19‐CAR‐DNTs can be stored in liquid nitrogen for up to 9 months to maintain their viability, % CAR^+^ cells and purity (CD3^+^CD4^−^CD8^−^). Remarkably, these cells also retain their cytotoxic activity against Raji cells (E: T = 4:1) during these storage periods (*n* = 3). Data represent three independent experiments using three different healthy donor‐derived CD19‐CAR‐DNT products, *P < 0.05 (*t*‐test). **(b)** Over 2 h after the cells were thawed at room temperature, the cryopreserved CD19‐CAR‐DNTs maintained their viability, % of CAR^+^ cells and purity, but the cytotoxic activity of the cells against Raji cells was best maintained for 1 h after thaw (*n* = 3). Data are shown as mean ± SD and are representative of three independent experiments using three different healthy donor‐derived CD19‐CAR‐DNT products, *P < 0.05, **P < 0.01 (*t*‐test).

Effective cryopreservation techniques offer significant advantages in adoptive cell therapy, enabling long‐term cell storage, ensuring product consistency and facilitating immediate infusion. Additionally, this technology facilitates the remote distribution of cell products, making this treatment modality accessible to a broader patient population. To evaluate the shelf life of cryopreserved CD19‐CAR‐DNTs, we analysed viability, purity, CAR^+^ cells % and antitumor activity of CD19‐CAR‐DNTs post‐cryopreservation up to 9 months in a liquid nitrogen tank. As depicted in Figure 3a, no significant decline in viability, CAR^+^ cells %, purity or antitumor activity was observed, confirming their stability and readiness for use even after 270 days of storage.

To further assess the bioactivity of the frozen CD19‐CAR‐DNTs at room temperature after thawing, we analysed their key biological characteristics over a period of 2 h, simulating clinical use conditions. As shown in Figure 3b, CD19‐CAR‐DNTs' viability, % of CAR^+^ cells and purity remained largely unchanged for 2 h. Although the antitumor activity of CD19‐CAR‐DNTs remained stable within 1 h after thawing (*P* > 0.05), it declined at 2 h (*P* < 0.05). Thus, it is necessary to administer the CD19‐CAR‐DNTs within 1 h of thawing.

### CAR‐DNTs display a less differentiated memory cell phenotype

The differentiation of T‐cell subsets plays a pivotal role in determining their longevity and replicative potential. T‐cell differentiation states include naive T cells or stem central memory T cells (T_N/SCM_, CD45RA^+^CD62L^+^), central memory T cells (T_CM_, CD45RA^−^CD62L^+^), effector memory T cells (T_EM_, CD45RA^−^CD62L^−^) and effector T cells (T_EFF_, CD45RA^+^CD62L^−^).^30^, ^31^ The phenotypic characteristics of less‐differentiated memory CAR‐Ts, specifically the proportion of T_SCM_ and T_CM_ cells, have been positively correlated with improved clinical outcomes in both preclinical and clinical investigations.^32^, ^33^

**Figure 4:**
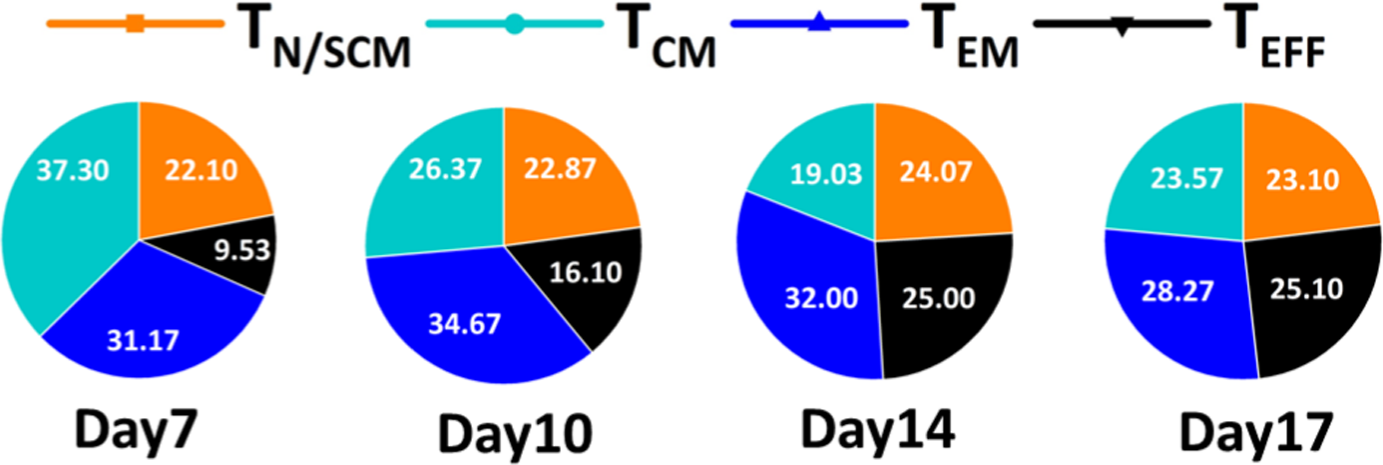
Flow cytometric analysis of the memory status of CD19 chimeric antigen receptor double‐negative T cells (CD19‐CAR‐DNTs) over 17 days from *ex vivo* expansion. In our research, memory cell subsets are defined by the expression of CD45RA and CD62L: naive or stem cell memory T cells (T_N/SCM_, CD45RA^+^CD62L^+^); central memory T cells (T_CM_, CD45RA^−^CD62L^+^), effector memory T cells (T_EM_, CD45RA^−^CD62L^−^) and effector T cells (T_EFF_, CD45RA^+^CD62L^−^). Pie charts show the composition of the different cell subtypes of CD19‐CAR‐DNTs at each time point during the 17‐day expansion period (*n* = 3). Data are shown as mean values of each % memory cell subset.

In our research, we characterised the expression of memory markers on CD19‐CAR‐DNTs and found that 40–50% of CD19‐CAR‐DNTs maintain a T_N/SCM_ and T_CM_ phenotype over a 17‐day culture period. Specifically, on Day 7, the average percentage of T_N/SCM_ and T_CM_ cells was 59.40%, remaining relatively stable at 46.67% (ranging from 37.2% to 64.2%) on Day 17 (Figure 4). Furthermore, CD19‐CAR‐DNTs exhibit low expression of the inhibitory receptor PD‐1 (CD279), Lag‐3 and the T‐cell ageing marker CD57. In contrast, they constitutively express high levels of Tim‐3 (CD336), consistent with previous reports (Table 2, *n* = 5).^28^ In conclusion, the maintenance of a high proportion of T_N/SCM_ and T_CM_ cells, along with their resistance to exhaustion and senescence, renders CAR‐DNTs a promising candidate for antitumor immunotherapy.

**Table 2:** Expression of exhaustion markers

	PD‐1 (%)	TIM‐3 (%)	Lag‐3 (%)	CD57 (%)
Healthy donor #1	1.20	72.80	32.10	47.60
Healthy donor #2	4.90	90.60	37.80	30.00
Healthy donor #3	3.60	80.30	17.70	22.50
Healthy donor #4	4.90	94.70	24.50	23.90
Mean ± SD	3.65 ± 1.74	84.60 ± 9.93	28.03 ± 8.78	31 ± 11.54

### CD19‐CAR‐DNTs exhibit enhanced DNT cytotoxicity *in vitro* and *in vivo*

**Figure 5:**
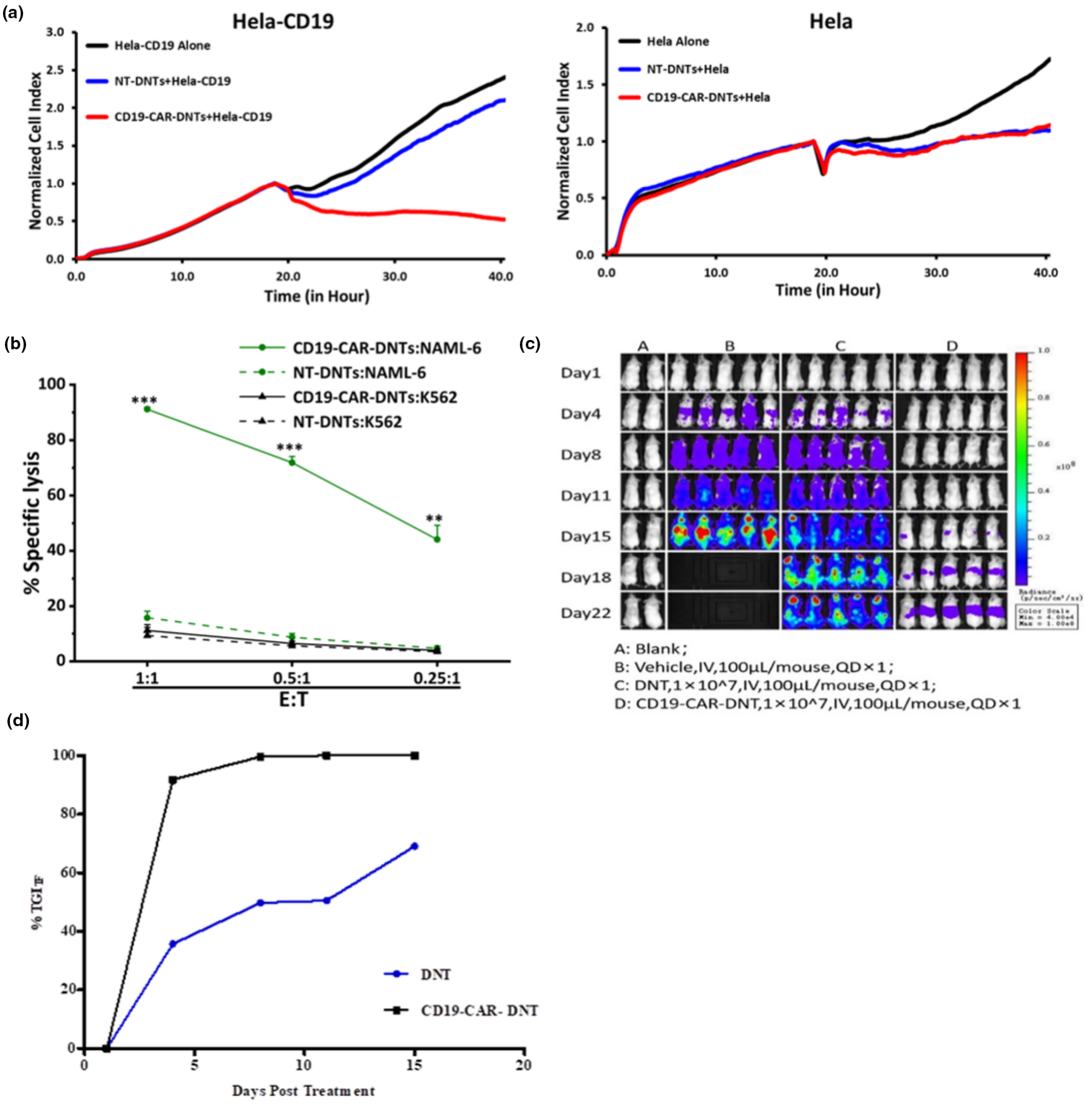
CD19 chimeric antigen receptor double‐negative T cells (CD19‐CAR‐DNTs) enhance the cytotoxic activity of non‐transduced double‐negative T cells (DNTs) *in vitro* and *in vivo*. **(a)** Long‐term cytotoxicity was assessed using the RTCA assay. CD19‐CAR‐DNTs and NT‐DNTs were incubated with Hela‐CD19 or Hela cells for 20–24 h at E:T ratios of 1:1. Representative results from three independent experiments using three different donor‐derived CD19‐CAR‐DNT products are shown. The cytotoxicity against target cells was monitored every 10 min over 22 h using the RTCA system. The cell index of target cells was used as a measure of cytotoxicity. Dots represent the mean percent specific killing of duplicates at E:T ratios of 1:1. **(b)** Short‐term cytotoxicity was assessed using flow cytometry. CD19‐CAR‐DNTs and NT‐DNTs were co‐cultured with PKH‐26 labelled NALM‐6 or K562 cells for 3 h at different E:T ratios (1:1, 0.5:1, 0.25:1). The percent specific killing of these targets was measured, representing the mean percent specific killing of triplicates, and error bars represent standard deviation. Experiments were independently performed three times from three different healthy donor‐derived CD19‐CAR‐DNT products. ***P < 0.001, **P < 0.01 (*t*‐test). **(c, d)** 6‐8 weeks female NOG mice were intravenously injected with 5 × 10^5^ Raji‐Luc cells. One day later, 10 × 10^6^ fresh double‐negative T cells (DNTs), CD19‐CAR‐DNTs, or vehicles were administered intravenously, equivalent to 5.4 × 10^6^ CAR^+^ cells/mouse. Bioluminescence images were acquired at Day 1, Day 4, Day 8, Day 11, Day 15, Day 18, Day 22. Mice were randomised into three groups based on their fluorescence intensity before CD19‐CAR‐DNTs injection. Imaging **(c)** and inhibition rate (%TGI_TF_) **(d)** of tumor cell growth of different treatments (*n* = 1–5).

To investigate the capacity of CD19‐CAR‐DNTs to enhance the cytotoxic activity of DNTs against CD19^+^ target cells, we conducted co‐culture experiments with Hela‐CD19 cells at E:T ratios of 2:1 for 22 h and NALM‐6 cells at various E:T ratios for 3 h (Figure 5a, b). CD19‐CAR‐DNTs exerted significantly superior killing of Hela‐CD19 cells and NALM‐6 cells compared with NT‐DNTs across all E:T ratios. CD19‐CAR‐DNTs were equally efficient as TN‐DNTs in killing K562 cells and Hela cells, indicating that CD19‐CAR‐DNTs showed enhanced specific cytotoxic activity by the CD19‐CAR receptor.

To validate these findings *in vivo*, we evaluated the antitumor activity of CD19‐CAR‐DNTs using a Raji‐Luc xenograft mouse model. One day after the mice were intravenously injected with 5 × 10^5^ Raji‐Luc cells, they were administered with a single intravenous infusion of 10 × 10^6^ fresh DNTs, CD19‐CAR‐DNTs or vehicle (five mice per group). Tumor progression was monitored by measuring changes in tumor bioluminescence over time, and the mice were killed on Day 22. While tumor bioluminescence rapidly increased in vehicle‐treated mice, both DNTs and CD19‐CAR‐DNTs infusions significantly inhibited tumor growth (Figure 5c). Notably, CD19‐CAR‐DNTs demonstrated a superior TGI rate of 99.98% on Day 15, compared with 69.04% for DNTs, underscoring their enhanced therapeutic potential (Figure 5d). Remarkably, this trend persisted even on Day 22, with CD19‐CAR‐DNTs maintaining a significantly higher level of tumor control compared to DNTs (*P* < 0.001).

### Large‐scale manufactured cryopreserved CD19‐CAR‐DNTs exhibit robust cytotoxicity against B‐cell malignancies *in vitro* and *in vivo*

**Figure 6:**
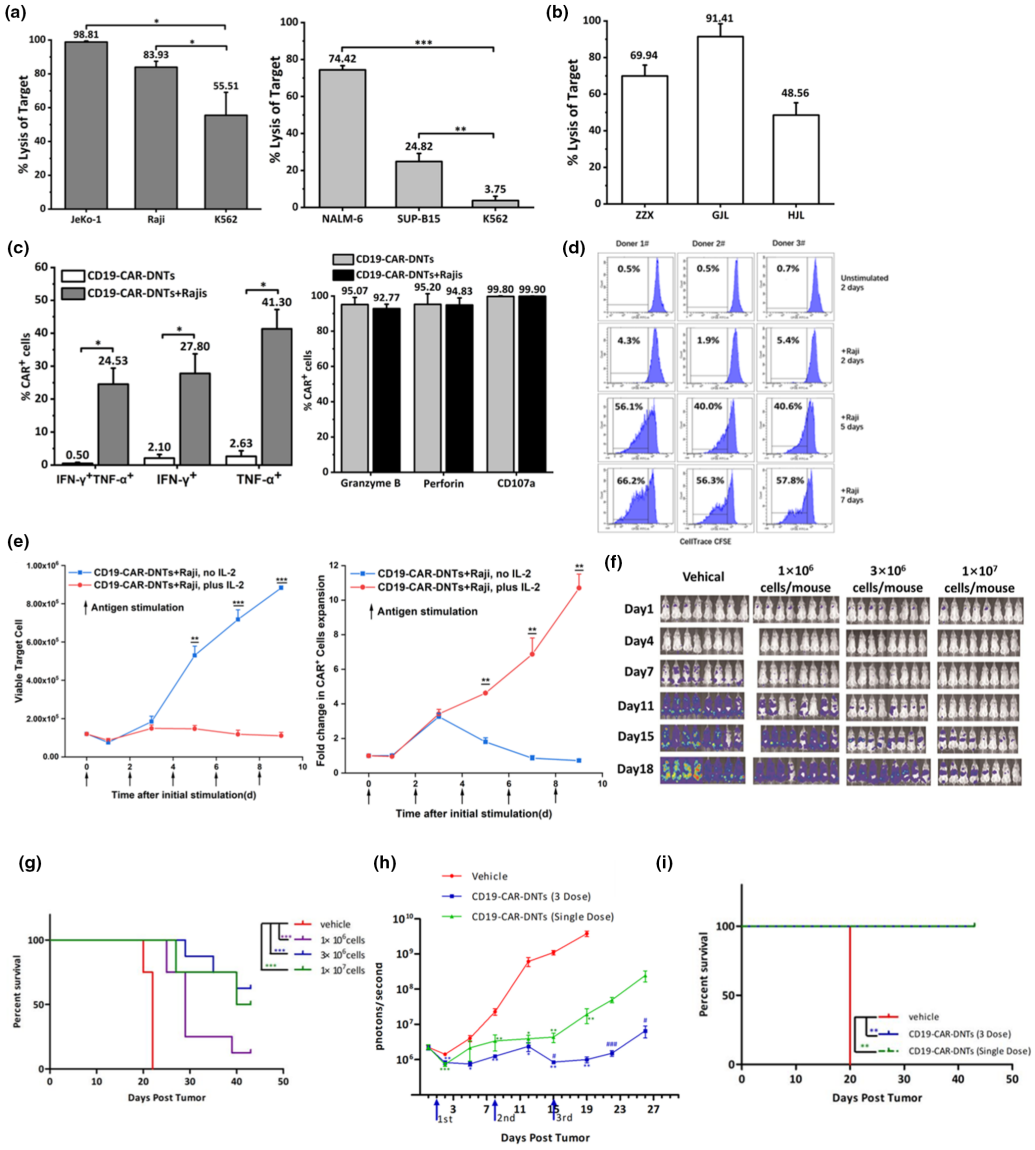
CD19 chimeric antigen receptor double‐negative T cells (CD19‐CAR‐DNTs) show robust effector functions against CD19^+^ tumor targets *in vitro* and *in vivo*. **(a)** CD19‐CAR‐DNTs induce superior cytotoxicity against B‐ALL cell lines (SUP‐B15, NALM‐6) at E: T ratio of 1:1 and B‐NHL cell lines (JeKo‐1, Raji) at E: T ratio of 4:1. CD19‐CAR‐DNTs were co‐cultured with PKH‐26 labelled target cells for 3 h; the per cent specific killing of the targets was measured. The results represent the mean percent specific killing of triplicates, and error bars indicate standard deviation (SD). Experiments were independently performed three times. *P < 0.05, **P < 0.01, ***P < 0.001. **(b)** CD19‐CAR‐DNTs were co‐cultured with primary B‐ALL blasts for 3 h at an effector‐to‐target (E:T) ratio of 5:1. The cytotoxicity was calculated using the formula: 100 × (%Annexin V^+^with CD3^−^CD10^+^ of co‐culture – %Annexin V^+^ with CD3^−^CD10^+^of primary B‐ALL blasts alone)/(100 – %Annexin V^+^with CD3^−^CD10^+^ of primary B‐ALL blasts alone). Data are shown as mean ± SD of triplicates and are representative of three independent experiments using cells from three different donors. **(c)** The expression of intracellular IFN‐γ, TNF‐α,107a, granzyme and perforin in CD19‐CAR‐DNTs was determined by flow cytometry in the absence or presence of Raji cells. The results are presented as mean ± SD of triplicates and are representative of three independent experiments. *P < 0.05 (*t*‐test). **(d)** The proliferative potential of CD19‐CAR‐DNTs following three rounds of antigen exposure was measured over a 7‐day culture period. Aliquots of cells harvested on Days 2, 5 and 7 were analysed by flow cytometry to determine cellular proliferation based on dye dilution. Representative data from three independent experiments using CD19‐CAR‐DNTs derived from three healthy donors are shown. % proliferation after each stimulation was calculated using a formula that accounts for proliferation in the presence or absence of Raji cells. **(e)** The potential of CD19‐CAR‐DNTs to proliferate and suppress tumor cell growth in the presence or absence of 250 IU human IL‐2 after repeated stimulation with Raji cells (E: T ratio of 1:2). Data are shown as the mean ± SD (*n* = 3). **(f)** *In vivo* efficacy of three different doses (1 × 10^6^ cells/mouse, 3 × 10^6^ cells/mouse, 10 × 10^6^ cells/mouse, equivalent to 0.6 × 10,^6^, 1.8 × 10^6^, 6.1 × 10^6^ CAR^+^ cells/mouse) of CD19‐CAR‐DNTs in Raji‐Luc xenograft model. Mice were randomised into four groups based on their fluorescence intensity before CD19‐CAR‐DNTs injection. Bioluminescence images were acquired on Days 1, 4, 8, 11, 15 and 18. **(g)** Kaplan–Meier curve showing the survival probability of Raji‐engrafted mice treated with vehicle (*n* = 5) or three dose levels of CD19‐CAR‐DNTs, ***P < 0.001. **(h)** Raji‐Luc‐xenografted NOG mice were administered with cryopreserved CD19‐CAR‐DNTs (10 × 10^6^ cells/mouse, equivalent to 5.2 × 10^6^ CAR^+^ cells/mouse) or vehicle either once or thrice intravenously (on days 1, 8 and 15). Bioluminescence images were acquired on Days 1, 2, 5, 8, 12, 15, 19, 22 and 26. Data represent the mean ± SD, where asterisks (*) indicate the comparison of single infusion (green) or multiple infusions (blue) with vehicle control, *P < 0.05, **P < 0.01, ***P < 0.001. Hash marks (#) indicate the comparison of multiple infusions (blue) with a single infusion, ^#^ *P* < 0.05, ^###^ *P* < 0.001. **(i)** Kaplan–Meier curve showing the survival probability of Raji‐engrafted mice treated with vehicle or single or multiple administrations, **P < 0.01.

To further explore the antitumor potential of large‐scale manufactured CD19‐CAR‐DNTs in targeting B‐cell malignancies, cryopreserved CD19‐CAR‐DNTs were co‐cultured with various CD19^+^ cell lines. Notably, CD19‐CAR‐DNTs elicited remarkable cytotoxicity against B‐NHL cell lines (Raji and JeKo‐1), B‐cell acute lymphoblastic leukaemia (B‐ALL) cell lines (NALM‐6 and SUP‐B15), and primary B‐ALL blasts (Figure 6a, b). Meanwhile, our results concurred with previous findings by Zhang *et al*. that CD19‐CAR‐DNTs exhibited comparable cytotoxic activity to CD19‐CAR‐Ts against Raji cells and Hela‐CD19 cells (Supplementary figure 3a–e).^28^ Next, we analysed the expression of key intracellular cytotoxic factors upon antigen stimulation (Figure 6c). Our findings revealed a marked upregulation of IFN‐γ and TNF‐α in CD19‐CAR‐DNTs following co‐cultured with Raji cells. Notably, CD19‐CAR‐DNTs expressed high levels of CD107a, perforin and granzyme B, both in the presence or absence of Raji cells, indicating their rapid response to antigens and inherent antitumor immunity. This cytokine profile was consistent with that of native DNTs, suggesting that CD19‐CAR‐DNTs may possess a favorable safety profile for clinical applications.^34^, ^35^ To further evaluate the proliferation and potential cytotoxicity of CD19‐CAR‐DNTs under repeated antigen stimulation, CFSE‐labelled CD19‐CAR‐DNTs were stimulated with irradiated Raji cells every 2 days. Compared with unstimulated cells, the proliferation of CD19‐CAR‐DNTs increased to 3.32 ± 1.72%, 39.59 ± 9.35% and 51.46 ± 2.83% on Days 2, 5 and 7, respectively. After the second and third rounds of stimulation, CD19‐CAR‐DNTs exhibited significantly higher proliferation, with dye dilution indicative of multiple rounds of cellular division observed in all three donor‐derived CD19‐CAR‐DNTs products (Figure 6d). Notably, in the presence of exogenous IL‐2, CD19‐CAR‐DNTs exhibit more robust recursive killing potential at high tumor cell loads, achieving durable TGI (Figure 6e).

The *in vivo* efficacy of CD19‐CAR‐DNTs was evaluated in a Raji‐Luc xenograft model. Mice were injected intravenously with 5 × 10^5^ Raji‐Luc cells. One day after the Raji‐Luc injection, cryopreserved CD19‐CAR‐DNTs or vehicles were administered intravenously at three dose levels. Tumor growth was tracked at regular intervals up to Day 18 by monitoring total bioluminescence flux (TF). As expected, TF increased sharply in vehicle‐treated mice, particularly after Day 7, while CD19‐CAR‐DNTs suppressed tumor burden in a dose‐dependent manner (Figure 6f) and significantly prolonged the survival compared with vehicle control (Figure 6g). To determine whether multiple infusions of the cells could further enhance the antitumor activity, tumor‐bearing NOG mice were administered with vehicle, cryopreserved CD19‐CAR‐DNTs either once or thrice intravenously (on Days 1, 8 and 15). Our results revealed that multiple administrations can further bolster the antitumor effect to inhibit tumor growth (Figure 6h, i).

### Pharmacokinetics of CD19‐CAR‐DNTs

**Figure 7:**
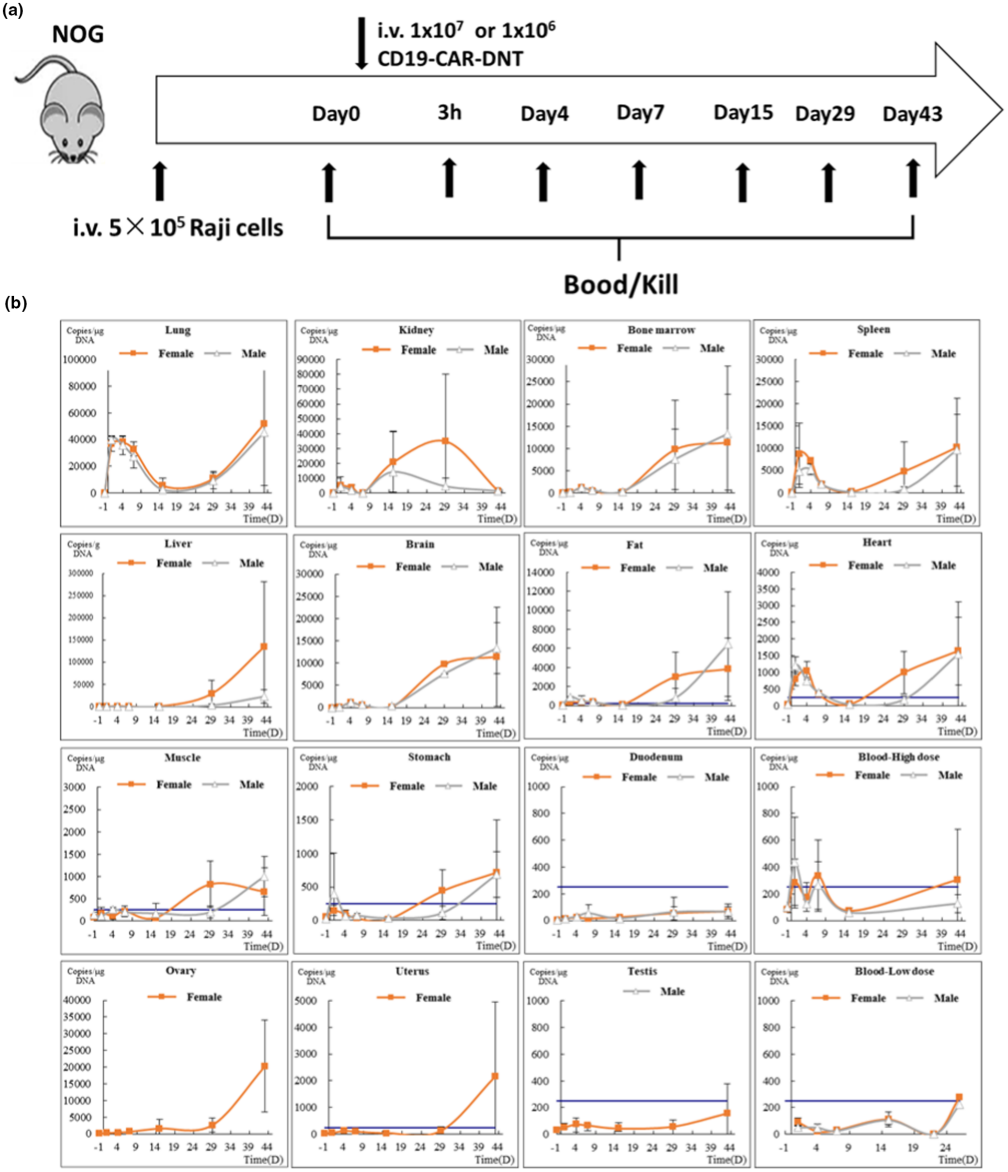
CD19 chimeric antigen receptor double‐negative T cells (CD19‐CAR‐DNTs) distribution and biodynamics in NOG mice. **(a)** Flow diagram of the experiment. NOG mice were injected intravenously via the tail vein with 5 × 10^5^ Raji‐Luc cells. One day later, cryopreserved CD19‐CAR‐DNTs were administered intravenously at two dose levels (1 × 10^6^ cells/mouse or 10 × 10^6^ cells/mouse). Ten mice (five of each gender) were killed at each time point (0 h, 3 h, Day 4, Day 7, Day 15, Day 29, Day 43) after CD19‐CAR‐DNTs infusion. Blood and organs were collected to detect CAR gene copies and cytokine profiles. In the high‐dose group, both blood and organs were collected, while in the low‐dose group, only blood was collected. **(b)** Changes in the CAR gene in the organs and blood over time were measured. The blue line represents the lower limit of quantification. Data are presented as mean ± SD.

To elucidate the biodistribution, proliferation and persistence of large‐scale manufactured CD19‐CAR‐DNTs *in vivo*, we administered 1 × 10^6^ or 10 × 10^6^ cryopreserved CD19‐CAR‐DNTs to Raji‐Luc tumor‐bearing NOG mice. Over the course of the study, tissues and blood were harvested to analyse CD19‐CAR copies on Days 1, 4, 7, 15, 29 and 43 (Figure 7a).

In peripheral blood, CAR gene copies were observed in a dose‐dependent manner within three hours post infusion; subsequently, it exhibited a decreasing or fluctuating trend and maintained low levels. Notably, CAR gene copies were detected in all tissues except the testes and duodenum. Three hours after administration, CD19‐CAR‐DNTs were widely distributed in the organs well‐perfused with blood, including the lung, spleen, kidney, heart and liver, with the lung showing the highest level. Over time, the CAR copies exhibited a decreasing or fluctuating trend initially, but from Day 15 to 43, there was a marked increase in CAR gene content, peaking at Day 43, indicating that the activation and amplification of CD19‐CAR‐DNTs appeared in most of the tissues (Table 3, Figure 7b). The area under the curve (AUC) showed that CD19‐CARs were primarily distributed in the lung and liver, followed by the kidney, bone marrow, ovary, spleen, fat, brain, heart, muscle and stomach (Table 3).

**Table 3:** Summary of CAR‐DNT parameters in tissues and whole blood (mean)

Tissue	Dose (cells/mouse)	Sex	Tmax (days)	Cmax (Copies/μg DNA)	AUC (d*Copies/μg DNA)
Lung	10 × 10^6^	Male	43	52 065	947 766
		Female	43	45 138	807 190
Liver		Male	43	135 545	1 380 609
		Female	43	23 587	228 677
Bone marrow		Male	43	11 397	224 837
		Female	43	13 291	211 026
Duodenum		Male	43	66	1671
		Female	43	67	1942
Muscle		Male	29	821	18 785
		Female	43	1003	14 017
Brain		Male	43	10 168	79 020
		Female	43	4725	36 404
Spleen		Male	43	10 171	189 584
		Female	43	9583	116 066
Blood		Male	7	335	8512
		Female	1	433	5573
Kidney		Male	29	34 989	753 001
		Female	15	14 265	241 921
Stomach		Male	43	719	12 170
		Female	43	686	8190
Heart		Male	43	1639	32 605
		Female	43	1544	20 755
Fat		Male	43	3826	74 141
		Female	43	6476	62 644
Testis		Male	43	159	3156
Ovary		Female	43	20 283	201 092
Uterus		Female	43	2163	18 143
Blood	1 × 10^6^	Male	26	280	3038
		Female	26	223	2609

### CD19‐CAR‐DNTs significantly suppress tumor growth and effectively ameliorate the tissue damage caused by tumor cells *in vivo*

To assess the cytotoxicity of large‐scale manufactured CD19‐CAR‐DNTs, 120 NOG mice (60 non‐tumor‐bearing, 60 tumor‐bearing) were used. Tumor‐bearing mice received Raji‐Luc cells. Both tumor‐bearing and non‐tumor‐bearing mice were further randomised into subgroups (15/sex/group) based on weight and treated with CD19‐CAR‐DNTs (10 × 10^6^ cells/mouse, equivalent to 6.1 × 10^6^ CAR^+^ cells/mouse) or vehicle (freezing buffer). Mice were monitored for 6 weeks and killed on Day 15 or 43 for evaluation.

**Figure 8:**
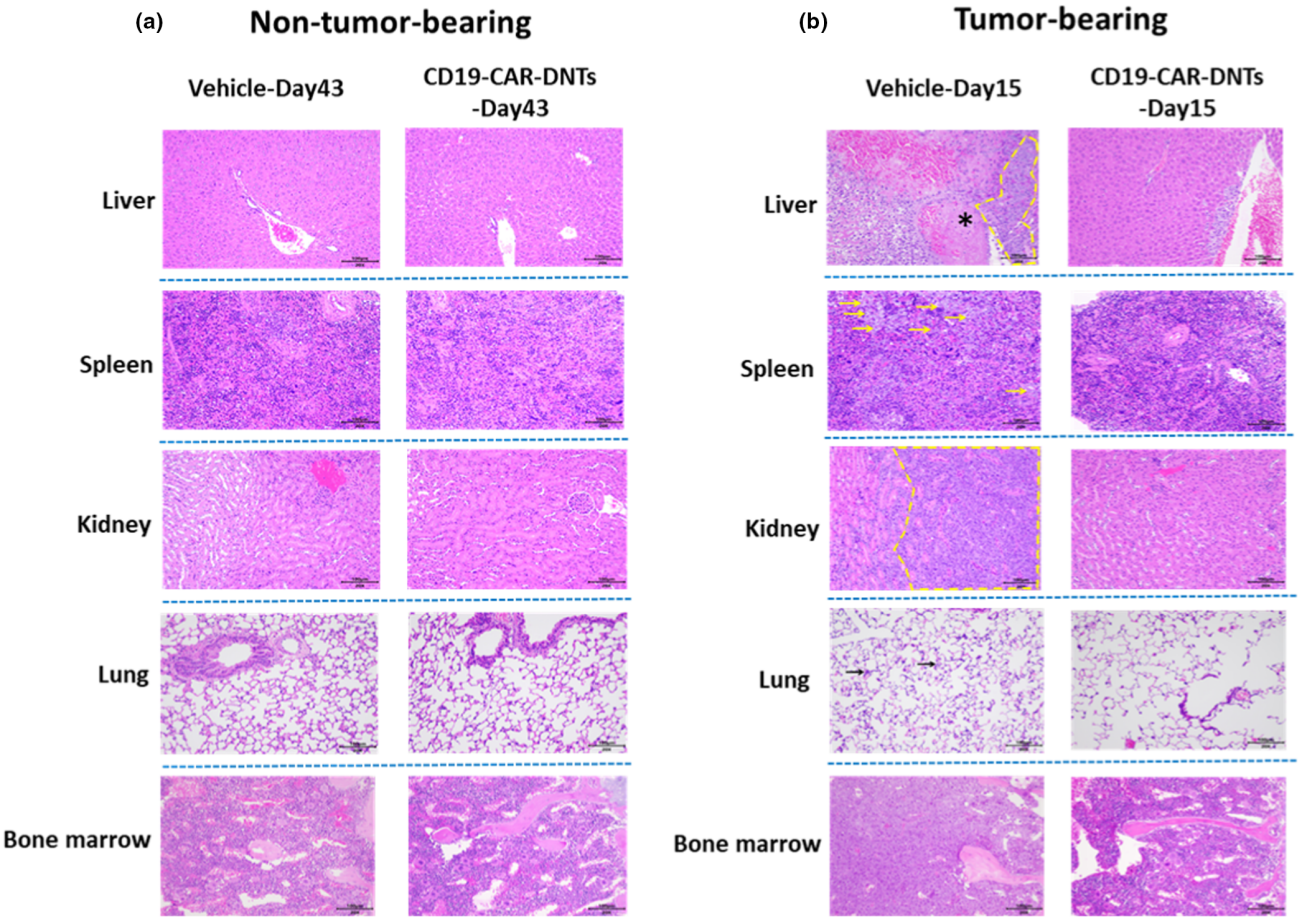
CD19 chimeric antigen receptor double‐negative T cells (CD19‐CAR‐DNTs) are safe and can effectively ameliorate the tissue damage caused by tumor cells in NOG mice. **(a)** Representative images (×20 magnification) of hematoxylin and eosin (H&E)‐stained slides from the liver, spleen, kidney, lung and bone marrow of non‐tumor‐bearing mice on Day 43 post administration are shown. Compared to those receiving freezing buffer alone mice (Left), no significant pathological changes or signs of toxicity were observed in mice treated with CD19‐CAR‐DNTs (Right). **(b)** Representative images (×20 magnification) of H&E‐stained slides from the liver, spleen, kidney, lung and bone marrow of tumor‐bearing mice. Lymphoma was observed in the liver, spleen, kidney, lung and bone marrow in Raji‐xenografted mice treated with vehicle on Day 15. This was characterised by the presence of large‐sized, strongly basophilic, round or irregular‐shaped lymphoma cells with prominent nucleoli (indicated by yellow or black arrows or yellow dashed boxes) and fibrosis in the liver (indicated by asterisks) (Left). On Day 15, the CD19‐CAR‐DNT‐treated mice revealed a notable reduction in lymphoma infiltration and improved tissue morphology compared to vehicle‐treated controls (Right).

By Day 43, the vehicle‐treated tumor‐bearing group had a mortality rate of 100% (20/20), while the CD19‐CAR‐DNT‐treated group had 25% (5/20). Notably, in the non‐tumor‐bearing mice, no abnormal clinical symptoms, pathological changes or deaths were observed throughout the study period, regardless of whether they were treated with CD19‐CAR‐DNTs or freezing buffer (Supplementary tables 1–4, Figure 8a). This indicates that CD19‐CAR‐DNTs are safe and have no cytotoxicity in non‐tumor‐bearing mice.

In the tumor‐bearing mice, lymphoma was observed, attributed to the xenografting of Raji‐Luc cells, and appeared as white spots in the liver, spleen and kidney and as nodules in the uterine and skin groyne. Additionally, enlargement of the ovaries and pituitary was noted at necropsy (Supplementary tables 1 and 2). On Day 15 after freezing buffer administration, tumor‐bearing mice exhibited widespread lymphoma infiltration in nearly all organs except the eyes. In contrast, mice treated with CD19‐CAR‐DNTs displayed a significantly reduced lymphoma infiltration with some mononuclear CD19‐CAR‐DNTs infiltration into the tumor sites (Supplementary table 3, Figure 8b). On Day 43, surviving CD19‐CAR‐DNT‐treated tumor‐bearing mice exhibited increased lymphoma infiltration accompanied by pathological changes, as shown in supplementary table 4. These changes were attributed to the heavy tumor burden. Additionally, mononuclear cell infiltration was prominent in multiple organs, particularly in the liver.

In conclusion, CD19‐CAR‐DNTs demonstrate remarkable safety in non‐tumor‐bearing mice, causing no observed toxicity or GvHD. Moreover, CD19‐CAR‐DNTs have shown remarkable efficacy in suppressing tumor growth and reducing tumor‐induced tissue damage in a xenograft model.

## Discussion

Previous studies have demonstrated that DNTs fulfil all the requirements for off‐the‐shelf allogeneic cell therapy, including expansion capacity, antitumor activity, ready‐to‐use, no GvHD and HvGR.^18^, ^25^ DNTs target a range of hematologic and solid cancers *in vitro* and *in vivo*. Clinical trials have demonstrated the safety and potential efficacy of healthy unrelated donor‐derived allogeneic DNTs as a treatment for relapsed and refractory AML patients.^26^, ^27^ Here, we further evaluated the feasibility, preclinical toxicity, efficacy and tissue distribution of using allogeneic CAR‐DNTs as an off‐the‐shelf therapy platform. We engineered humanised CD19‐ScFv fragments into second‐generation CAR structures and transduced DNTs derived from healthy donors using a lentiviral vector. We found that DNTs could be stably transduced with the lentiviral vector, and the transduction did not alter DNTs' innate characteristics. We developed a large‐scale manufacturing process that expands CD19‐CAR‐DNTs to clinically relevant numbers, achieving up to an average of 2977.5‐fold expansion after 14 days of culture *in vitro*. Moreover, CD19‐CAR‐DNTs can be stored in liquid nitrogen for 270 days under GMP conditions without hampering their biofunction.

The phenotypic characteristics of less‐differentiated memory CAR‐Ts have been positively correlated with improved outcomes in both preclinical and clinical investigations.^30^, ^31^, ^32^, ^33^ In our study, we demonstrated that despite robust proliferation, CD19‐CAR‐DNTs exhibited a high purity of CD3^+^CD4^−^CD8^−^ T cells, strong cytotoxic activity and low expression levels of differentiation and exhaustion markers. *In vitro* cytotoxicity assay, CD19‐CAR‐DNTs displayed strong cytotoxicity against CD19^+^ cell lines and primary lymphoblasts from the patient. Furthermore, CD19‐CAR‐DNTs possessed higher proliferative potential even after multiple rounds of tumor antigen stimulations. As confirmed by the previous study, CD19‐CAR‐DNTs exhibited comparable cytotoxic activity to CD19‐CAR‐Ts against Raji cells and Hela‐CD19 cells.^28^ DNTs exert moderate cytotoxicity mediated by non‐specific innate receptors, such as NKG2D and DNAM‐1, which bind to corresponding ligands expressed on cancer cells. However, their specific cytotoxic activity is further augmented by the CAR, therefore enhancing its antitumor activity. Since CD19‐CAR‐DNTs act as a ‘living drug’, their initial behaviour, organ distribution and targeting after the infusion are critical. In the preclinical assessments, we demonstrated that CD19‐CAR‐DNTs inhibited tumor growth in a dose‐dependent manner via a single intravenous administration in the B‐NHL xenograft model; multiple administrations further augment the antitumor effect. Activation and amplification of CD19‐CAR‐DNTs were observed in most tissues. CD19‐CAR‐DNTs were widely distributed in the well‐perfused organs initially, extensively spreading to most organs in 2–3 weeks and peaking at Day 43 after the administration. We observed a peak in CAR gene copy numbers on Day 43, with no decrease over the 43‐day study. This suggests that further investigation of the long‐term biodynamics of CD19‐CAR‐DNTs is warranted. In conclusion, CD19‐CAR‐DNTs can specifically target CD19^+^ tumor cells *in vitro* and *in vivo*, with the capacity to proliferate and persist in tumor‐infiltrated organs.

Many studies have shown that mechanisms of action by DNTs to kill AML or lung cancer cell lines via various innate receptors, such as NKG2D and DNAM‐1, and releasing cytotoxic factors such as IFN‐γ and TNF‐α, thereby making target cells more susceptible to their cytotoxic activity.^14^, ^17^, ^24^ Consistent with the innate antitumor immunity of DNTs, CD19‐CAR‐DNTs displayed high levels of CD107a, perforin and granzyme B, even in the absence of Raji cells, indicative of their inherent antitumor immunity and rapid response to antigenic challenges. Furthermore, our findings revealed a marked upregulation of IFN‐γ and TNF‐α in CD19‐CAR‐DNTs following co‐culture with Raji cells. In the absence of target cells, CAR‐DNTs express modest levels of intracellular IFN‐γ and TNF‐α, whereas CAR‐Ts express high levels of these cytokines, suggesting that CD19‐CAR‐DNTs may possess a favorable safety profile for clinical applications. Cytokines such as IL‐2 and IL‐15 are vital for augmenting the *in vivo* proliferation and antitumor activity of T cells and NK cells.^16^, ^35^, ^36^ In our study, exogenous IL‐2 was crucial for supporting the proliferation of CD19‐CAR‐DNTs, achieving durable TGI *in vitro*. In addition to the well‐established role of type 1 cytokines, recent findings suggest a beneficial character of type 2 cytokines, such as IL‐10 or IL‐4, in promoting antitumor effects.^37^, ^38^, ^39^ These cytokines modulate the metabolic activities of CAR‐Ts to resist their dysfunction, leading to tumor eradication and long‐lasting immune protection. Further studies are necessary to explore this strategy to enhance CAR‐DNT efficacy in clinical application.

Although autologous CAR‐Ts against haematological tumors have achieved deep and durable responses with manageable safety, there is a pressing need for allogeneic CAR immune cell therapies that are safe, effective and affordable. However, GvHD and persistence in the host remain major concerns in developing off‐the‐shelf CAR‐T products.^40^, ^41^ TCR knockout helps avoid GvHD, but it increases cost, manufacturing complexity and the potential risk of incomplete knockout or chromosomal variations.^9^, ^40^, ^41^ HvGR is a major extrinsic factor affecting allogeneic cell persistence that is highly correlated with antitumor response.^40^, ^41^ Strategies such as TCR/HLA depletion and preconditioning aim to avoid HvGR, but NK cell‐mediated rejection remains a challenge.^42^, ^43^, ^44^ Clinical trials have demonstrated that allogeneic DNT therapy is a safe and effective treatment option for relapsed and refractory AML patients: 10 patients received three planned doses of allogeneic DNTs administered weekly, and no signs of GvHD or severe neurotoxicity were observed.^26^, ^27^ CAR‐DNTs further enhance the cytotoxic activity of DNTs through CAR while maintaining the same treatment safety *in vivo*. Studies have shown that no GvHD or other treatment‐related toxicity was observed in mice infused with CD19‐CAR‐DNTs, whereas infusion of CD19‐CAR‐Ts induced GvHD in six of seven NSG mice irradiated with a sublethal dose.^28^ Moreover, it was found that, unlike CD19‐CAR‐Ts, CD19‐CAR‐DNTs are more resistant to HvGR and evade allogeneic T‐cell alloreactivity by actively suppressing the priming of allogeneic CD8^+^ T cells to allogeneic antigens *in vitro*.^28^ In this study, a comprehensive preclinical safety assessment of CD19‐CAR‐DNTs was performed using NOG mice, and it was further confirmed that no signs of immunotoxicity or GvHD were observed in the non‐tumor‐bearing mice treated with CD19‐CAR‐DNTs.

In summary, we have successfully developed a novel immunotherapy approach using engineered DNTs derived from healthy donors. Specifically, DNTs were effectively transduced using a lentiviral vector to manufacture CD19‐CAR‐DNTs. These engineered cells can be cryopreserved for long periods and exhibit remarkable efficacy against CD19^+^ lymphoma both *in vitro* and *in vivo*. In preclinical studies using NOG mice, CD19‐CAR‐DNTs demonstrated the ability to be activated and amplified without causing toxicity or GvHD. Overall, CAR‐DNTs represent a promising platform for allogeneic CAR‐T cell therapy targeting various hematologic and solid malignant tumors as well as some autoimmune diseases. Our team has initiated a phase I clinical study to evaluate the safety and efficacy of RJMty19, an allogeneic CD19‐CAR‐DNT cell product, in the treatment of relapsed and refractory B‐NHL (NCT06314828) and systemic lupus erythematosus (NCT06340490).

## Methods

### Cell lines

Raji cells (American Type Culture Collection, Gaithersburg, MD, USA) and Jeko‐1 cells (American Type Culture Collection) were cultured and expanded in RPMI 1640 medium (ThermoFisher Scientific, Waltham, MA, USA) supplemented with 10% FBS (ExCell Bio., Suzhou, China). SUP‐B15 cells (BeNa Culture Collection, Henan, China) and NALM ‐6 cells (BeNa Culture Collection) were cultured and expanded in RPMI 1640 medium (ThermoFisher Scientific) supplemented with 10% FBS (ExCell Bio.) and 1% sodium pyruvate (ThermoFisher Scientific). K562 cells (ATCC) were cultured and expanded in IMDM (ThermoFisher Scientific) medium supplemented with 10% FBS (ExCell Bio.). Hela cells (ATCC) were cultured and expanded in DMEM (ThermoFisher Scientific) medium supplemented with 10% fetal bovine serum (FBS) (ExCell Bio.). Hela‐CD19 cells were a gift from ProMab Biotechnologies, Inc., and were cultured and expanded in DMEM (ThermoFisher Scientific) medium supplemented with 10% FBS (ExCell Bio.) and 1 μg mL^−1^ puromycin (Selleckchem, Shanghai, China). All the cell lines mentioned above were incubated in a 37°C and 5% CO_2_ incubator. These cell lines were used as target cells in the *in vitro* cytotoxicity experiments. All cell lines were authenticated by STR profiling (ATCC or Genetic testing Bio., Suzhou, China) and tested negative for mycoplasma contamination.

### Membrane Proteome Array

Membrane proteome array (MPA) screening was conducted by Integral Molecular, Inc. (Shanghai, China). The MPA is a protein library composed of 6000 distinct human membrane protein clones, each overexpressed in live cells from expression plasmids. Each clone was individually transfected into separate wells of a 384‐well plate followed by a 24‐h incubation (Tucker *et al*. 2018).^45^ Cells expressing each MPA protein clone were arrayed in duplicate in a matrix format for high‐throughput screening. Before screening on the MPA, the test ligand concentration for screening was determined on cells expressing positive (membrane‐tethered protein A) and negative (mock‐transfected) binding controls, followed by flow cytometry detection using a fluorescently labelled secondary antibody. Each test ligand was added to the MPA at the predetermined concentration (20 μg mL^−1^), and binding across the protein library was measured on an Intellicyt iQue (Sartorius, Gottingen, Germany) using a fluorescently labelled secondary antibody (3.75 μg mL^−1^, 1:400). Each array plate contains both positive (Fc‐binding) and negative (empty vector) controls to ensure plate‐by‐plate reproducibility. Test ligand interactions with any targets identified by MPA screening were confirmed in a second flow cytometry experiment using serial dilutions of the test antibody, and the target identity was re‐verified by sequencing.

### Construction of the humanised CD19‐CAR Lentiviral vector and CD19‐CAR transduction

We constructed the humanised CD19 scFv from a mouse CD19 FMC63 scFv clone, containing a mutation in CDR1 of VH (V27G in VH) (PCT/CN2019/109633). We generated a humanised CD19‐ScFv‐CAR construct under the MNDU3 promoter inside the lentiviral vector. The lentiviral CAR construct contains a CD8 hinge, CD8 trans‐membrane, 4‐1BB co‐stimulatory domain and CD3ζ signalling domains. For CD19‐CAR‐DNTs production, PBMCs were collected using Ficoll‐Paque Plus (STEMCELL Technologies, Cambridge, MA, USA). To enrich DNT cells, CD4^+^ and CD8^+^ T cells together with red blood cells were depleted using RosetteSep® depletion kit according to the manufacturer's instruction (STEMCELL Technologies). CD4^+^ and CD8^+^ cell‐depleted healthy donor‐derived PBMCs were collected and washed with 0.9% saline. This method successfully isolated NDTs with > 85% purity, and DNTs were cultured with anti‐CD3 antibody (GMP‐grade OKT3; Miltenyi Biotec, Auburn, CA, USA) for activation. At 24–48 h after activation, DNTs were transduced with CD19 lentivirus (MOI = 1–10) and cultured in a CO_2_ incubator. Fresh medium was added to the cultures every 1–2 days; the transduced DNTs were continuously cultured for 10–14 days *ex‐vivo*; then, the cells were harvested, cryopreserved and ready for use after thaw. The manufacture of CD19‐CAR‐DNT production has been authorised by an international patent (PCT/CN2021/087311).

### Manufacturing of clinical‐grade self‐inactivating lentiviral vectors

The manufacture of GMP‐grade lentiviruses were manufactured by GenScript ProBio (Nanjing, China), which has established GMP capacity that meets the regulatory requirements of the Chinese National Medical Products Administration (NMPA) and the US Food and Drug Administration (FDA).

### Cryopreservation of CD19‐CAR‐DNTs

CD19‐CAR‐DNTs are formulated in CryStor CS5 freezing media (BIOLIFE, Bothell, WA, USA), frozen using a cryopreservation device and stored in liquid nitrogen tanks for use. The CD19‐CAR‐DNTs were thawed in a 37°C‐water bath for 3–5 min.

### Flow cytometry

The following anti‐human antibodies were utilised in our experiments: CD3, CD4, CD5, CD8, CD16, CD56, CD14, CD20, TCR γ/δ, TCR Vδ2, CD57, CD62L, CD45RA, 7‐AAD, CD197 (CCR7), CD279 (PD‐1), CD366 (TIM3) and CD223 (LAG‐3), all sourced from Biolegend (San Diego, CA, USA). Additionally, TCR α/β was procured from BD Bioscience (San Jose, CA, USA), while TCR Vδ1 was obtained from Ebiosciences (ThermoFisher Scientific). The expression of ScFv‐CAR was quantified utilising PE streptavidin and biotinylated anti‐mouse FMC63 antibodies from BIOSWAN (Shanghai, China). Samples were acquired on a BD FACSCanto™ II (BD Biosciences) flow cytometer. Data were analysed using the FlowJo™ software.

### Flow cytometry‐based cytotoxicity assay

To evaluate the cytotoxicity of fresh or cryopreserved CD19‐CAR‐DNTs, they were co‐cultured with PKH‐26 (Sigma, St Louis, MO, USA) labelled target cells at different E:T ratios in 1640 medium (ThermoFisher Scientific) containing 10% FBS (ExCell Bio.) for 3 h. Subsequently, cells were stained with annexin V (Biolegend) or/and anti‐human CD3 (Biolegend) and anti‐human CD10 (Biolegend) and analysed by flow cytometry. The formula for calculating cytotoxicity is as follows: 100 × (%Annexin V^+^_with CD19‐CAR‐DNT_ – %Annexin V^+^_without CD19‐CAR‐DNT_) / (100 – %Annexin V^+^ _without CD19‐CAR‐DNT_).

The killing of primary cells from patients was performed following the method described by Vasic *et al*.^28^

### Real‐time cellular Analyser‐based cytotoxicity assay

The cytotoxicity of CD19‐CAR‐DNTs was also evaluated by using xCELLigence RTCA (Real‐Time Cellular Analyser) (Agilent, Santa Clara, CA, USA). Fresh or cryopreserved CD19‐CAR‐DNTs were co‐cultured with target cells using a titrated E:T ratios for 20–24 h after pre‐incubation of 5000 target cells for at least 16‐20 h in 1640 medium (ThermoFisher Scientific) containing 10% FBS (ExCell Bio). The cytotoxicity against target cells was monitored every 10 min over 22–24 h using the RTCA system. The cell index of target cells was used as a measure of cytotoxicity. To calculate the cytotoxicity, the following formula was used: (Target cell alone normalised cell index – Target cell with effector cell normalised cell index) / (Target cell alone normalised cell index) × 100. The ‘Normalized cell index’ refers to the relative cell growth or impedance value obtained from the RTCA instrument for each condition.

### Antigen stimulation and proliferation of CAR‐DNTs

As antigen for stimulation, Raji cells were irradiated with 6000 cGy. CD19‐CAR‐DNTs were labelled with CFSE (Biolegend) and then plated alone or with Raji cells at a 1:1 ratio for 2–3 days per stimulation in a serum‐free culture medium. Samples were harvested on the 2nd, 5th and 7th day, respectively, which were analysed by flow cytometry to assess proliferation by dye dilution. % proliferation was calculated using the following formula: 100 × (% Proliferated with Raji – % Proliferated without Raji) / (100 – % Proliferated without Raji).

### Tumor cell rechallenge assay

The long‐term cytotoxic function and proliferative capacity of CD19‐CAR‐DNTs after repetitive target antigen engagement was evaluate as previously reported.^46^ Briefly, CD19‐CAR‐DNTs were co‐cultured with Raji cells at a 1:2 ratio, in the presence or absence of 250 IU human IL‐2, to evaluate CD19‐CAR‐DNT's recursive killing potential under high tumor cell loads. In this assay, the long‐term cytotoxic function and proliferative capacity of CAR‐DNTs are examined *in vitro* over 9 days with addition of initial double amount of Raji cells to the co‐culture every other day.

### Intracellular cytokine detection assay

CD19‐CAR‐DNTs (1 × 10^5^ cells/well) were co‐cultured with Raji cells at a ratio of 1:1 for 3 h. Negative control wells contained only CD19‐CAR‐DNTs or Raji cells, while positive control wells contained CD19‐CAR‐DNTs and 0.01 μL of cell activation cocktail (Biolegend). The Golgiplug™ and fixation/permeabilisation reagent set from BD Biosciences was used for intracellular staining. The following anti‐human antibodies were used for cell staining: CD3‐FITC, CD107a‐PE/Cy7, perforin‐APC/Cyanine7, granzyme B‐PE, INF‐γ‐APC/Cyanine7 and TNF‐α‐PE, and these were purchased from Biolegend. Data acquisition was performed using a BD FACSCanto™ II (BD Biosciences). Flow cytometry data were analysed using the FlowJo™ software.

### Primary cell samples from patients

The primary cell samples from B‐ALL patients were collected with informed consent and were approved by the Ethics Committee of the Second Affiliated Hospital, Zhejiang University School of Medicine. Primary cells were isolated via density gradient centrifugation using Ficoll‐Paque Plus (STEMCELL Technologies). The isolated primary cells were cryopreserved in a storage medium containing 90% FBS (ExCell Bio.) and 10% DMSO (Sigma).

### *In vivo* studies

To evaluate the long‐term cell persistence, safety and antitumor efficacy of cryopreserved CD19‐CAR‐DNTs *in vivo*, we employed an immunodeficient NOG (NOD.Cg‐Prkdcscid Il2rgtm1Sug/JicCrl) (Charles River Co., Ltd., Beijing, China) xenograft model. All animal experiments were conducted with the approval of the Institutional Animal Care and Use Committee (IACUC) and adhered to the ‘Guidelines for Good Laboratory Practice for Non‐clinical Laboratory Studies’ issued by the National Medical Products Administration (NMPA) in 2017. Strict adherence to the standard operating procedures and experimental protocols of their research institution was ensured.

### Efficacy studies

Female NOG mice 6–8 weeks of age were intravenously injected with 5 × 10^5^ Raji‐Luc cells. One day later, various dosages of cryopreserved CD19‐CAR‐DNTs or vehicles were administered either once or thrice times intravenously (on Days 1, 8 and 15). Bioluminescence images were captured and analysed using the IVIS Imaging System (PerkinElmer, Waltham, MA, USA).

### Pharmacokinetic studies

NOG mice were intravenously injected with 5 × 10^5^ Raji‐Luc cells. One day later, mice were randomised into two groups based on their fluorescence intensity. Subsequently, cryopreserved CD19‐CAR‐DNTs were administered intravenously at two dose levels (1 × 10^6^ cells/mouse or 10 × 10^6^ cells/mouse) to assess cell expansion, persistence and tissue distribution. Mice (five of each gender per time point) were sampled on Day 1 (3 h post dosing) and Days 4, 7, 15, 29 and 43. For the qPCR assay to detect CD19‐CAR copies, DNA from different tissues and blood was extracted using an E.Z.N.A Tissue DNA Kit (OMEGA, Norcross, GA, USA) following the manufacturer's instructions, and DNA concentrations were quantified using UV spectrophotometry (Implen, Munich, Germany) and adjusted to a suitable concentration range. Primers and probes for CAR‐DNTs were designed and synthesised by GenScript Biotech (Nanjing, China). The amount of DNA fragment from each sample was quantitatively measured on Applied Biosystems®7500 fast Real‐time PCR systems (ThermoFisher Scientific). These data were processed using 7500 Fast system 21 CFR Part 11 Module SDS software Version 1.5.1.

### *In vivo* safety and toxicity studies

To assess the cytotoxicity of CD19‐CAR‐DNTs, 120 NOG mice (60 non‐tumor‐bearing and 60 tumor‐bearing) were used. Tumor‐bearing mice received Raji‐Luc cells. Both tumor‐bearing and non‐tumor‐bearing mice were further randomised into subgroups (15/sex/group) based on their body mass and were treated with CD19‐CAR‐DNTs (10 × 10^6^ cells/mouse, equivalent to 6.1 × 10^6^ CAR^+^ cells/mouse) or vehicle. Mice were monitored for 6 weeks and killed on Day 15 or 43. Five mice of each gender were killed on Day 15, and on Day 43, ten mice of each gender were sacrificed for necropsy. Eyeballs, harderian glands, optic nerves and testes were fixed in modified Davidson's fixative, while the heart, lungs, liver, spleen, kidneys, brain, epididymis, ovaries, uterus, abdomen skin, injection site (tail) and other tissues were fixed in 10% formalin and histopathological examination was carried out by qualified pathologists.

### Statistics

Data in results are expressed as means ± standard deviations (SD) *in vitro*. Analyses were performed with Origin Pro, and the statistical significance was determined using two‐tailed unpaired or paired Student's *t‐*tests. *P < 0.05, **P < 0.01, ***P < 0.001 and ****P < 0.0001 indicate significance between experimental and control values. Data represent the means ± SD of *n* values *in vivo*, where *n* corresponds to the number of mice used. Statistical analyses were performed using one‐way ANOVA, followed by Dunnett's test for comparisons against the untreated or buffer group. Analyses were performed with GraphPad Prism 7 (version 7.0), and the statistical significance was determined using SPSS, as values were considered significantly different when *P* < 0.05. Pharmacokinetic parameters were calculated based on a non‐compartmental mode by using the WinNonlin 8.0 software.

## Author contributions

DW, LW, SL and LY were involved in conception and design. LW, JT, HZ, MX and XL performed the experiment and/or development of methodology. ZX and QS conducted large‐scale CD19‐CAR‐DNTs preparation. HW performed the quality assurance of the products. DW, LW and SL analysed and interpreted the data. YW and SW performed the *in vivo* experiments and conducted data analysis. DW and LY were involved in writing, review and/or revision of the manuscript.

## Author contributions

**Dan Wang:** Conceptualization; investigation; methodology; project administration; writing – original draft. **Liuyang Wang:** Conceptualization; data curation; formal analysis; investigation; methodology; software; visualization. **Shuai Liu:** Data curation; formal analysis; methodology; software. **Jianjun Tong:** Data curation; formal analysis; investigation; methodology. **Honglin Zhu:** Data curation; methodology; resources. **Man Xu:** Formal analysis; methodology; resources. **Xiancai Li:** Data curation; methodology. **Zhiqiang Xiang:** Methodology; resources. **Qinghua Sun:** Methodology; resources. **Hengcai Wang:** Supervision; validation. **Yuli Wang:** Data curation; methodology; resources. **Shuyang Wang:** Data curation; methodology; resources. **Liming Yang:** Conceptualization; funding acquisition; project administration; writing – review and editing.

## Conflict of interest

DW, LW, SL, JT, HZ, MX, XL, ZX, QS, HW and LY are employed by Wyze Biotech Co. Ltd, Zhongshan, Guangdong, China. DW, LW and LY are inventors of several DNT technology‐related patents and intellectual properties. The remaining authors declare no competing interests.

## Supporting information


Supplementary figure 1.
Supplementary figure 2.
Supplementary table 1.
Supplementary table 2.
Supplementary table 3.

## Data Availability

The data that support the findings of this study are available upon request from the corresponding author [LY] upon reasonable request.
