# Supplementary material for: A preclinical study of allogeneic CD19 chimeric antigen receptor double‐negative T cells as an off‐the‐shelf immunotherapy drug against B‐cell malignancies
Source: Clin Transl Immunology. 2024 Dec 24;13(12):e70022. doi: 10.1002/cti2.70022 (PMC11667769; doi:10.1002/cti2.70022)
Supplement: Supplementary file 1 — Supplementary figure 1. Supplementary figure 2. Supplementary table 1. Supplementary table 2. Supplementary table 3. [file CTI2-13-e70022-s001.docx]

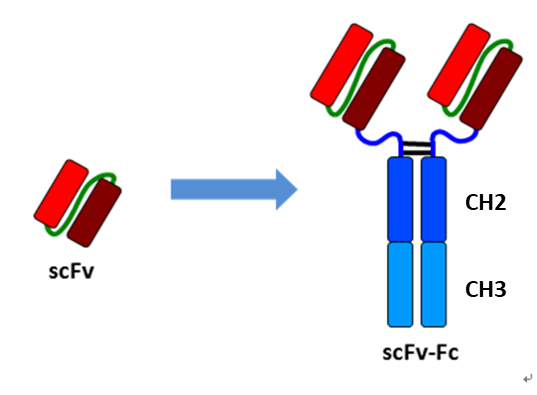


**Supplementary figure 1.** Schematic diagram of anti-human CD19 minibody. The anti-human CD19 minibody was constructed by fusing the CD19 scFv to the hinge region, CH2 and CH3 domain of mouse IgG2a to form a bivalent scFv molecule.


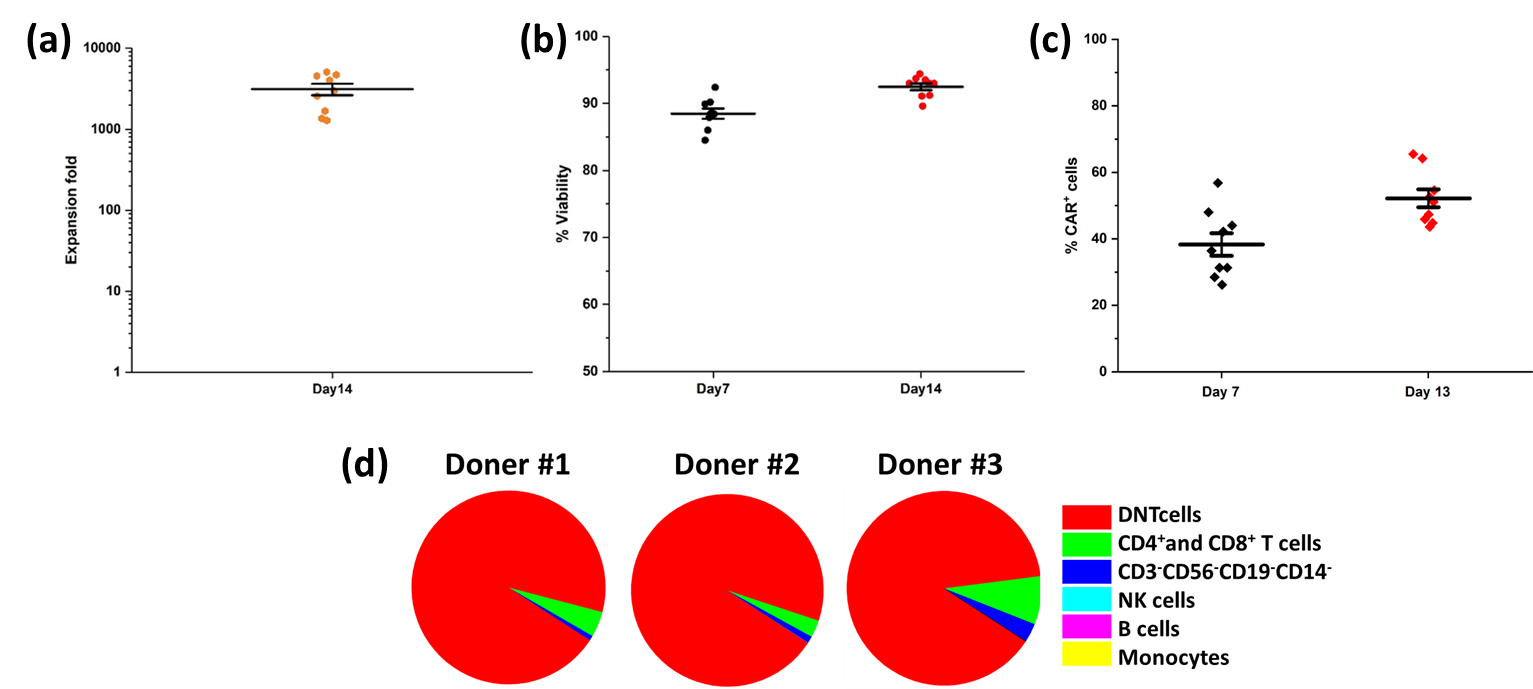


**Supplementary figure 2.** CD19-CAR-DNTs can be manufactured on a large scale under GMP conditions. The CD19-CAR-DNTs was manufactured using DNTs derived from healthy donors (large-scale, n = 9). **(a)** Expanded folds of CD19-CAR-DNTs on Day 7, **(b)** Viability CD19-CAR-DNTs on Day 7 and Day 14 days. **(c)** Summary of CD19 expression level from large-scale manufacturing runs as measured by flow cytometry on Day 7 and Day 13. the results represent nine independent experiments using nine different donor-derived CD19-CAR-DNTcell products. Horizontal lines represent the mean, and the error bars represent SD. **(d)** The cellular composition of the final cell product was assessed by flow cytometry. Pie charts represent the composition of three representative donor-derived CD19-CAR-DNTs batches.


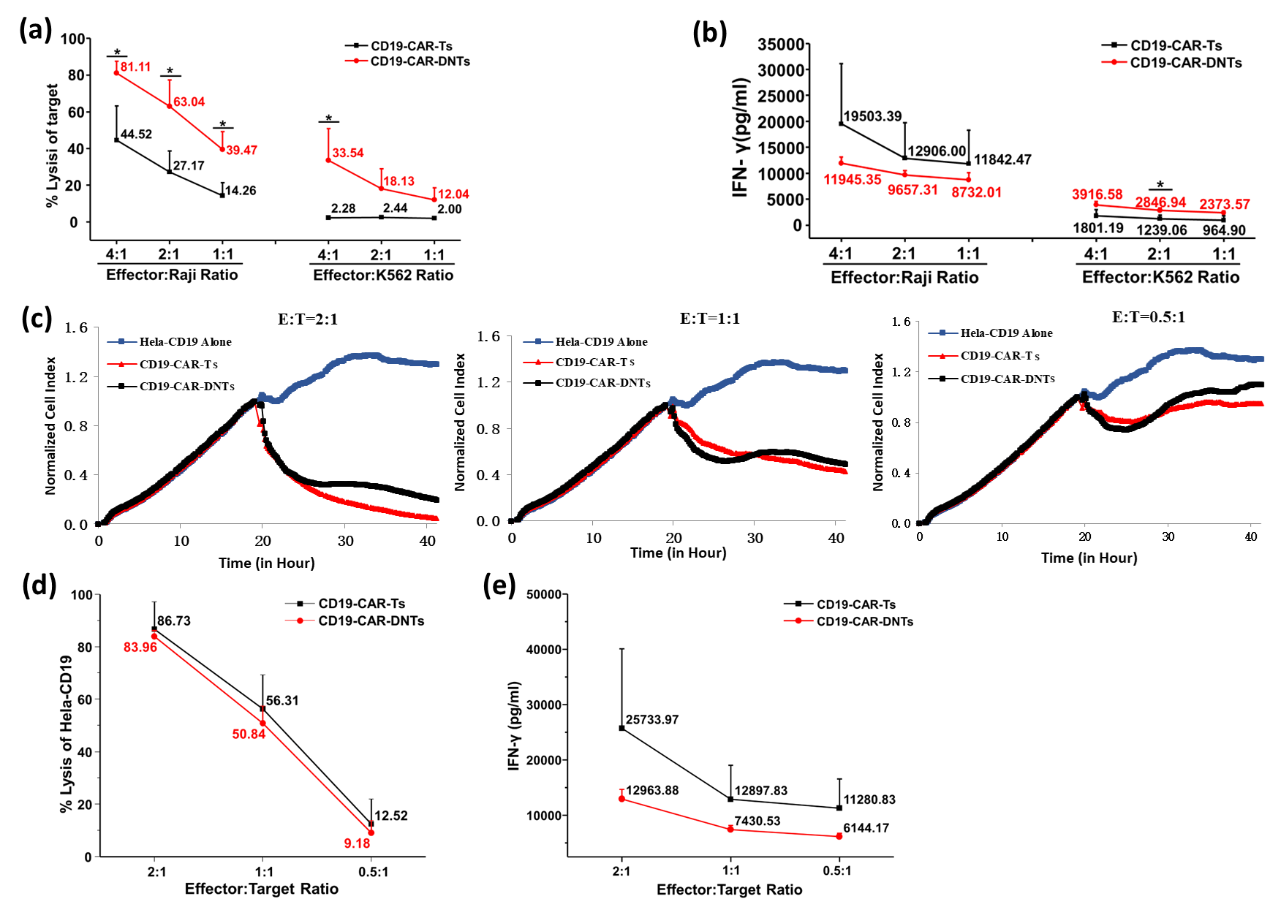


**Supplementary figure 3.** **Comparable cytotoxic activity of CD19-CAR-DNTs and CD19-CAR-Ts against CD19^+^ target cells in vitro. (a)** The mean percentage of specific killing by CD19-CAR-DNTs and CD19-CAR-Ts against Raji cells and K562 cells was determined over three hours at E:T ratios of 4:1, 2:1, and 1:1. **(b)** The resulting supernatant was collected to determine the concentration of IFN-γ. The data points represent the mean of triplicates, with error bars indicating SD, and are representative of three independent experiments, * *P*＜0.05. **(c)** To evaluate the durability of the cytotoxic effect, a 22-24-hour RTCA assay was performed using Hela-CD19 target cells at E:T ratios of 2:1, 1:1, and 0.5:1. The representative results from three independent experiments using three different donor-derived CD19-CAR-DNTcell products are shown. The cytotoxicity against target cells, reported as the normalized cell index, was monitored every 10 minutes throughout the assay. Dots represent the mean percent specific killing of duplicates. **(d)** At 20 hours of co-culture with effector cells, the cytotoxicity was calculated using the formula: (Target cell Alone Normalized cell index - Target cell with effector cell Normalized cell index) / (Target cell Alone Normalized cell index) × 100). **(e)** The levels of INF-γ secreted during the RTCA assay was measured by ELISA. Data are presented as mean ± SD of triplicates and representative of three independent experiments.

**Supplementary table 1.** The gross morphological changes-Day 15

| **Sex** | **male** | | | | | **female** | | | | |
| --- | --- | --- | --- | --- | --- | --- | --- | --- | --- | --- |
| **Tumor-bearing/**  **non-tumor-bearing** | **Non-tumor-bearing** | | **Tumor-bearing** | | | **Non-tumor-bearing** | | **Tumor-bearing** | | |
| **Administration** | **vehicle** | **CD19-CAR-DNTs** | **vehicle** | | **CD19-CAR-DNTs** | **vehicle** | **CD19-CAR-DNTs** | **vehicle** | | **CD19-CAR-DNTs** |
| **Dose（×10^6^ cells/mouse）** | **0** | **10** | **0** | **10** | | **0** | **10** | **0** | **10** | |
| **Number of mice** | **5** | **5** | **5** | **5** | | **5** | **5** | **5** | **5** | |
| **Liver（With the gallbladder）** | | | | | | | | | | |
| allochroic, white, diffuse | 0 | 0 | 5 | 0 | | 0 | 0 | 5 | 0 | |
| **Spleen** | | | | | | | | | | |
| allochroic, white, diffuse | 0 | 0 | 0 | 0 | | 0 | 0 | 1 | 0 | |
| **Ovary** | | | | | | | | | | |
| Enlarged, unilateral /bilateral | / | / | / | / | | 0 | 0 | 3 | 0 | |

/：NA (not applicable);

**Supplementary table 2.** The gross morphological changes-Day 43

| **Sex** | **male** | | | **female** | | |
| --- | --- | --- | --- | --- | --- | --- |
| **Tumor-bearing/non-tumor-bearing** | **Non-tumor-bearing** | | **Tumor-bearing** | **Non-tumor-bearing** | | **Tumor-bearing** |
| **Administration** | **vehicle** | **CD19-CAR-DNTs** | **CD19-CAR-DNTs** | **vehicle** | **CD19-CAR-DNTs** | **CD19-CAR-DNTs** |
| **Dose（×10^6^ cells/mouse）** | **0** | **10** | **10** | **0** | **10** | **10** |
| **Number of mice** | **10** | **10** | **9** | **10** | **10** | **6** |
| **Liver（With the gallbladder**） | | | | | | |
| Allochroic, white, diffuse | 0 | 0 | 1 | 0 | 0 | 0 |
| Protrusions, white, multifocality | 0 | 0 | 8 | 0 | 0 | 6 |
| **pituitary** | | | | | | |
| enlarged | 0 | 0 | 0 | 0 | 0 | 1 |
| **Uterus** | | | | | | |
| Protrusions, Translucent, unilateral /bilateral | / | / | / | 0 | 0 | 2 |

/：NA (not applicable);

**Supplementary table 3****.** Histopathological changes-Day 15

| **Sex** | **male** | | | | **female** | | | |
| --- | --- | --- | --- | --- | --- | --- | --- | --- |
| **Tumor-bearing/non-tumor-bearing** | **Non-tumor-bearing** | | **Tumor-bearing** | | **Non-tumor-bearing** | | **Tumor-bearing** | |
| **Administration** | **vehicle** | **CD19-CAR-DNTs** | **vehicle** | **CD19-CAR-**  **DNTs** | **vehicle** | **CD19-CAR-DNTs** | **vehicle** | **CD19-CAR-DNTs** |
| **Dose（×10^6^ cells/mouse）** | **0** | **10** | **0** | **10** | **0** | **10** | **0** | **10** |
| **Number of mice** | **5** | **5** | **5** | **5** | **5** | **5** | **5** | **5** |
| **Organ-Infiltration with lymphoma** | | | | | | | | |
| **Liver** | | | | | | | | |
| Slight or mild | 0 | 0 | 0 | 4 | 0 | 0 | 2 | 4 |
| Moderate or severe | 0 | 0 | 5 | 1 | 0 | 0 | 3 | 0 |
| Total incidence | 0 | 0 | 5 | 5 | 0 | 0 | 5 | 4 |
| **salivary gland, submandibular gland** | | | | | | | | |
| Slight or mild | 0 | 0 | 1 | 0 | 0 | 0 | 0 | 1 |
| **Stomach** | | | | | | | | |
| Mild to moderate | 0 | 0 | 3 | 0 | 0 | 0 | 0 | 0 |
| **Large intestine, Colon** | | | | | | | | |
| Mild | 0 | 0 | 0 | 0 | 0 | 0 | 1 | 0 |
| **Kidney** | | | | | | | | |
| Mild | 0 | 0 | 4 | 1 | 0 | 0 | 0 | 0 |
| **Sternum, Bone marrow** | | | | | | | | |
| Mild to severe | 0 | 0 | 5 | 0 | 0 | 0 | 4 | 0 |
| **Spleen** | | | | | | | | |
| Mild to severe | 0 | 0 | 4 | 0 | 0 | 0 | 4 | 0 |
| **Heart** | | | | | | | | |
| Slight | 0 | 0 | 1 | 0 | 0 | 0 | 0 | 0 |
| **Lung** | | | | | | | | |
| Slight or mild | 0 | 0 | 5 | 4 | 0 | 0 | 3 | 1 |
| **Pituitary** | | | | | | | | |
| Mild to moderate | 0 | 0 | 3 | 1 | 0 | 0 | 0 | 0 |
| **Adrenal** | | | | | | | | |
| Slight or mild | 0 | 0 | 5 | 1 | 0 | 0 | 5 | 0 |
| **Brain** | | | | | | | | |
| Mild to moderate | 0 | 0 | 2 | 0 | 0 | 0 | 0 | 1 |
| **Eye** | | | | | | | | |
| Moderate | 0 | 0 | 0 | 0 | 0 | 0 | 0 | 0 |
| **Femur** | | | | | | | | |
| Mild or moderate | 0 | 0 | 5 | 0 | 0 | 0 | 5 | 0 |
| **Sternum** | | | | | | | | |
| Mild or moderate | 0 | 0 | 1 | 0 | 0 | 0 | 5 | 0 |
| **Ovary** | | | | | | | | |
| Mild or moderate | / | / | / | / | 0 | 0 | 3 | 0 |
| **Stomach** | | | | | | | | |
| Atrophy: Gastric glands | | | | | | | | |
| Mild | 0 | 0 | 2 | 0 | 0 | 0 | 0 | 0 |
| Single-cell necrosis: Gastric glands, Epithelium | | | | | | | | |
| Mild | 0 | 0 | 1 | 0 | 0 | 0 | 0 | 0 |
| **Liver** | | | | | | | | |
| Infiltrated: mononuclear cells, lymphoma | | | | | | | | |
| Slight | 0 | 0 | 0 | 1 | 0 | 0 | 0 | 2 |
| Infarction | | | | | | | | |
| Mild or moderate | 0 | 0 | 3 | 0 | 0 | 0 | 0 | 0 |
| Thrombus: Fibrosis | | | | | | | | |
| Mild | 0 | 0 | 1 | 0 | 0 | 0 | 0 | 0 |
| **Kidney** | | | | | | | | |
| Denaturation: Renal tubular | | | | | | | | |
| Moderate | 0 | 0 | 0 | 1 | 0 | 0 | 0 | 0 |
| Atrophy: Focal, Renal tubular | | | | | | | | |
| Moderate | 0 | 0 | 0 | 1 | 0 | 0 | 0 | 0 |
| **Sternum, Bone marrow** | | | | | | | | |
| Decreased of cells: Hematopoietic cells | | | | | | | | |
| Mild | 0 | 0 | 1 | 0 | 0 | 0 | 0 | 0 |
| Infarction | | | | | | | | |
| Severe | 0 | 0 | 0 | 0 | 0 | 0 | 4 | 0 |
| Increased ratio of granulocytes to erythrocytes | | | | | | | | |
| Moderate | 0 | 0 | 0 | 1 | 0 | 0 | 0 | 0 |
| **Spleen** | | | | | | | | |
| Single-cell necrosis | | | | | | | | |
| Mild | 0 | 0 | 2 | 0 | 0 | 0 | 0 | 0 |
| Increased extramedullary hematopoiesis | | | | | | | | |
| Slight | 0 | 0 | 0 | 1 | 0 | 0 | 0 | 0 |
| **Femur** | | | | | | | | |
| Hyperostosis | | | | | | | | |
| Slight or mild | 0 | 1 | 1 | 0 | 0 | 0 | 1 | 0 |

/：NA (not applicable);

**Supplementary table 4.** Histopathological changes- Day 43

| **Sex** | **male** | | | **female** | | |
| --- | --- | --- | --- | --- | --- | --- |
| **Tumor-bearing/non-tumor-bearing** | **Non-tumor-bearing** | | **Tumor-bearing** | **Non-tumor-bearing** | | **Tumor-bearing** |
| **Administration** | **vehicle** | **CD19-CAR-DNTs** | **CD19-CAR-DNTs** | **vehicle** | **CD19-CAR-DNTs** | **CD19-CAR-DNTs** |
| **Dose（×10^6^ cells/mouse）** | **0** | **10** | **10** | **0** | **10** | **10** |
| **Number of mice** | **10** | **10** | **9** | **10** | **10** | **6** |
| **Organ-Infiltration with lymphoma** | | | | | | |
| **Liver** | | | | | | |
| Moderate or severe | 0 | 0 | 9 | 0 | 0 | 6 |
| Salivary gland, submandibular gland | | | | | | |
| Mild to moderate | 0 | 0 | 0 | 0 | 0 | 2 |
| **Stomach** | | | | | | |
| Mild to severe | 0 | 0 | 5 | 0 | 0 | 1 |
| **Kidney** | | | | | | |
| Slight or mild | 0 | 0 | 1 | 0 | 0 | 1 |
| **Sternum, Bone marrow** | | | | | | |
| Mild to moderate | 0 | 0 | 2 | 0 | 0 | 5 |
| **Lung** | | | | | | |
| Slight or mild | 0 | 0 | 6 | 0 | 0 | 4 |
| **Pituitary** | | | | | | |
| Mild to severe | 0 | 0 | 1 | 0 | 0 | 2 |
| **Adrenal** | | | | | | |
| Slight | 0 | 0 | 4 | 0 | 0 | 3 |
| **Brain** | | | | | | |
| Slight or mild | 0 | 0 | 4 | 0 | 0 | 4 |
| **Bone marrow（Neck, chest, waist）** | | | | | | |
| Slight | 0 | 0 | 1 | 0 | 0 | 0 |
| **Ovary** | | | | | | |
| Moderate | / | / | / | 0 | 0 | 2 |
| **Uterus** | | | | | | |
| Moderate or severe | / | / | / | 0 | 0 | 2 |
| **Cervix** | | | | | | |
| Mild | / | / | / | 0 | 0 | 1 |
| **Oviduct** | | | | | | |
| Severe | / | / | / | 0 | 0 | 1 |
| **Skin（breast）** | | | | | | |
| Severe | 0 | 0 | 0 | 0 | 0 | 1 |
| **Infiltrated by mononuclear cells** | | | | | | |
| **Liver** | | | | | | |
| Mild or moderate | 0 | 0 | 9 | 0 | 0 | 6 |
| **Kidney** | | | | | | |
| Slight | 0 | 0 | 1 | 0 | 0 | 0 |
| **Brain** | | | | | | |
| Slight | 0 | 0 | 0 | 0 | 0 | 1 |
| **Ovary** | | | | | | |
| Mild | / | / | / | 0 | 0 | 1 |
| **Uterus** | | | | | | |
| Slight | / | / | / | 0 | 0 | 1 |
| **Oviduct** | | | | | | |
| Mild | / | / | / | 0 | 0 | 1 |
| **Stomach** | | | | | | |
| Slight or mild | 0 | 0 | 1 | 0 | 0 | 1 |
| Salivary gland, submandibular gland | | | | | | |
| Slight | 0 | 0 | 0 | 0 | 0 | 1 |
| **Liver** | | | | | | |
| Infarction | | | | | | |
| Mild or moderate | 0 | 0 | 3 | 0 | 0 | 3 |
| Fibrosis | | | | | | |
| Mild | 0 | 0 | 4 | 0 | 0 | 4 |
| **Stomach** | | | | | | |
| Atrophy: Gastric gland | | | | | | |
| Slight or mild | 0 | 0 | 2 | 0 | 0 | 1 |
| Single-cell necrosis: Increased in adenogastric epithelium | | | | | | |
| Mild | 0 | 0 | 1 | 0 | 0 | 0 |
| Erosion/ulceration | | | | | | |
| Slight or mild | 0 | 0 | 2 | 3 | 3 | 2 |
| Infiltrated: Mixed inflammatory cells, glandular gastric mucosa/ lymphoma | | | | | | |
| Slight or mild | 0 | 0 | 1 | 0 | 0 | 1 |
| **Sternum, Bone marrow** | | | | | | |
| Decreased of cells: Hematopoietic cells | | | | | | |
| Mild to moderate | 0 | 0 | 3 | 0 | 0 | 1 |
| **Spleen** | | | | | | |
| Infiltrated: mononuclear cells | | | | | | |
| Slight or mild | 0 | 0 | 4 | 0 | 0 | 1 |
| Increased extramedullary hematopoiesis | | | | | | |
| Slight or mild | 0 | 0 | 3 | 0 | 0 | 5 |
| Single cell necrosis | | | | | | |
| Mild | 0 | 0 | 3 | 0 | 0 | 4 |

/：NA (not applicable)；
